# Impact of a Natural Fusarial Multi-Mycotoxin Challenge on Broiler Chickens and Mitigation Properties Provided by a Yeast Cell Wall Extract and a Postbiotic Yeast Cell Wall-Based Blend

**DOI:** 10.3390/toxins14050315

**Published:** 2022-04-28

**Authors:** Manoj B. Kudupoje, Venkataramaiah Malathi, Alexandros Yiannikouris

**Affiliations:** 1 Alltech, Inc., 3031 Catnip Hill Road, Nicholasville, KY 40356, USA; ayiannikouris@alltech.com; 2 Poultry Science Department, Karnataka Veterinary, Animal and Fisheries Sciences University, Hebbal, Bengaluru 560024, India; drmalathiprasadreddy@rediffmail.com

**Keywords:** *Fusarium* mycotoxins, mycotoxin mitigation, yeast cell wall extract, postbiotic yeast cell wall-based blend, poultry broilers, EPEF

## Abstract

Yeast cell wall-based preparations have shown efficacy against *Aspergillus*-based toxins but have lower impact against type-B trichothecenes. Presently, we investigated a combination of deoxynivalenol (DON), T-2 toxin (T2) and zearalenone (ZEA), and the effect of a yeast cell wall extract (YCWE) and a post-biotic yeast cell wall-based blend (PYCW) with the objectives of preventing mycotoxins’ negative effects in commercial broilers. A total of 720 one-day-old male Cobb broilers were randomly allocated to: (1) control diet, (aflatoxins 6 µg/kg; cyclopiazonic acid 15 µg/kg; fusaric acid 25 µg/kg; fumonisin B1 310 µg/kg); (2) Diet1 + 0.2% YCWE; (3) Diet1 + 0.2% PYCW; (4) Contaminated diet (3.0 mg/kg DON; 2.17 mg/kg 3-acetyldeoxynivalenol; 104 g/kg T2; 79 g/kg ZEA); (5) Diet4 + 0.2% YCWE; and (6) Diet4 + 0.2% PYCW. Naturally contaminated diets adversely affected performance, serum biochemistry, liver function, immune response, altered cecal SCFA goblet cell count and architecture of intestinal villi. These adverse effects were reduced in birds fed PYCW and to a lesser extent YCWE, indicating protection against toxic assault. PYCW yielded better production performance and stimulated liver function, with higher response to NDV and IBV vaccination. Furthermore, mycotoxins were found to affect production outputs when evaluated with the European poultry production efficiency factor compared to control or YCWE and PYCW supplemented treatments. Taken together, YCWE, when complemented with nutritional add-ons (PYCW), could potentiate the remediation of the negative effects from a multi mycotoxins dietary challenge in broiler birds.

## 1. Introduction

Mycotoxins produced by several groups of fungi under favorable biotic and abiotic stress conditions [1,2,3] can have negative effects on livestock such as impaired kidney [4,5] and liver function [6,7,8], neurological impacts inducing emesis or anorexia [9,10], immunosuppression [11,12,13], growth retardation [14,15] and reproductive problems [16]. The simultaneous presence of different *Fusarium* fungal toxins along with *Aspergillus* and/or *Penicillium* spp. toxins could result in unexpected adverse effects on poultry productivity and health [17]. Even though feed companies follow strict guiding principles to keep toxin levels below the maximum permissible limits or recommended action levels or guidelines set by regulatory bodies (FDA and EU), the impact on animal performances due to interactions among different toxins is possible at much lower concentrations [18,19]. Results from recent survey work pinpoints the frequent co-occurrences of mycotoxins contaminating feed ingredients and compound feeds [17]. An analytical survey on corn-based feed collected between 2013–2019 identified an average of more than five different toxins out of the 35 tested per sample [20]. In addition, several toxins are identified by governing bodies as being responsible for health issues in poultry, with the maximum limit set to 5.0 mg/kg (total of type B trichothecenes including DON, 3-ADON, 15-acetyldeoxynivalenol (15-ADON), and DON-3-glucoside conjugate), 0.2 mg/kg (0.1 mg/kg in EU, including T-2 and HT2) and 0.4 mg/kg, respectively [18]. Therefore, managing mycotoxicosis represents an important priority for livestock producers globally, with the aim of maintaining productivity and animal health.

Yeast cell wall extracts (YCWE), at the inclusion rate of 0.025–0.15% exhibited some prebiotic functions when used in broiler diets, especially in overcoming immune stress induced by *Clostridium* infection [21], heat stress [22], or necrotic enteritis challenges [23]. Additionally, YCWE @ 1–2% in the diet enhanced broiler performance [24], by providing better antioxidant status [25], and could help counteract mycotoxicosis [26,27,28]. When used as a prebiotic, it has also shown positive effects in promoting the beneficial gut microbial population [29,30]. The skeletal structure of the yeast cell wall is made up from a highly ramified carbohydrate network and comprises mainly glucans, mannans and chitin, where the glucan fraction has been identified as the active component to zearalenone sequestration [31]. β-D-glucans from *Saccharomyces cerevisiae* cell wall have been extensively evaluated in-vitro [32,33], and has shown high adsorption affinity when tested at the rate of 0.1% *w*/*v*, towards several mycotoxins like aflatoxin B1, ochratoxin A, zearalenone, T-2 toxin and some of the toxins produced by *Penicillium* spp. [34,35]. However, other occurring mycotoxins including deoxynivalenol (DON), fumonisin B1 (FB1) and diacetoxyscirpenol (DAS) could be more challenging to interact with, due to their inherent chemical properties, larger amounts present in feed compared to other toxins and widespread chronic exposure. Nevertheless, in vivo work in broilers has extensively shown the benefit of YCWE (at 1–2 kg/T) at mitigating some of the effects induced by mycotoxins, especially from *Aspergillus,* but also *Fusarium* spp. [14,36,37,38,39,40]. In this context, it appeared important to evaluate if the benefit of a YCWE could be enhanced by the blending of carefully selected antioxidants and post-biotic fatty acids, constituting a postbiotic yeast cell wall-based blend (PYCW) to mitigate further the physiological effects in poultry broilers of a multi-mycotoxin challenge specifically involving *Fusarium* toxins.

Little is known about the beneficial effect of PYCW involving the interactions of several active constituents in addition to the YCWE and their complementary effect on the gastrointestinal environment and on different physiological processes. Our goal was to evaluate the cascading effects on animal health and productivity of those different components during a multi-mycotoxin stress that is matching or below the proposed regulatory guidelines defined earlier in the text. The main objective of this study was to evaluate the production performance, gut health and liver function parameters of broiler birds subjected to a mycotoxin challenge comprising a blend of grain-based laboratory produced *Fusarium* mycotoxins [41], evaluated with and against different dietary treatments. In addition, this study was designed to evaluate the potential beneficial effects of PYCW, compared to YCWE, in birds fed this naturally contaminated diet. The two major selected toxins identified by governing bodies [42,43,44] as responsible for health issues including DON and T-2 were targeted for this study.

## 2. Results

### 2.1. Performance: Body Weight, Feed Intake and Feed Conversion Ratio

The effects of DON contaminated diet and supplementation of YCWE and PYCW at the inclusion rate of 2.0 kg/T, on body weight (BW), feed intake (FI) and feed conversion ratio (FCR) calculated after correcting for mortality is presented in Table 1. The mortality rate was below 1.0% (7/720 birds) irrespective of treatment groups during the first two weeks of the study. Birds that died within two days of the start of the study (five birds) were replaced with two-day-old chicks to maintain an equal bird count throughout the experimental period.

In all weekly body weight measurements, as time progressed, a significantly lower BW (*p <* 0.018) was noticed for birds consuming a contaminated diet compared to a control diet (Table 1). Compared to their respective control (T1 or T4), unchallenged birds supplemented with PYCW (2 kg/T) exhibited greater BW on week four and week six, whereas higher body weight in birds fed contaminated diet was observed on the sixth week (*p <* 0.001). Additionally, at the end of the study, PYCW supplemented birds had better BW (*p <* 0.001) compared to YCWE supplemented unchallenged birds. Even though both YCWE and PYCW had higher BW compared to its challenged group (T4), comparing the supplemented birds, YCWE had higher BW than the PYCW group.

Feed intake was statistically identical across treatments, irrespective of the mycotoxin challenge or the supplementation of YCWE or PYCW at the end of the seven-weeks study (Table 1). However, during the third week, birds on the control diet showed higher FI (*p =* 0.002) compared to all other treatment groups.

Although supplementation had no impact on FI, the BW was higher in birds fed YCWE and PYCW, leading to a significantly lower FCR (Table 1). In birds fed a non-contaminated diet, YCWE and PYCW supplementation had lower FCR (*p =* 0.002) compared to its control diet on the third week of growth. Compared within the contaminated diet treatments, and contrasted with the contaminated diet treatment, both YCWE and PYCW supplemented birds exhibited better FCR during the sixth (*p* < 0.001) week of growth. Overall, at the end of the study, the FCR was lower with both YCWE and PYCW for non-challenged birds compared to its control T1, and also for challenged birds (*p* ≤ 0.001). Additionally, the FCR values of challenged birds receiving both supplements was similar to the value of the non-challenged control (T1).

### 2.2. Immunological Assessment: Lymphoid Organ Weight and Antibody Titers

**Figure 1:**
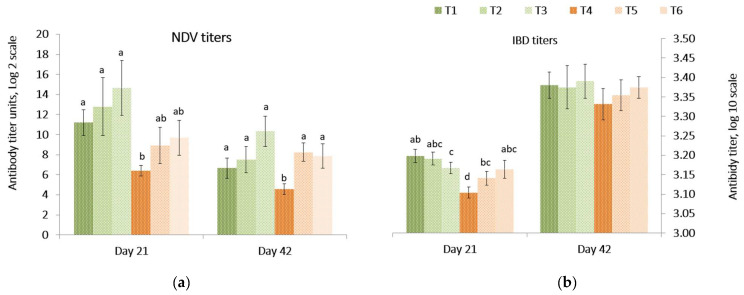
Effect of a *Fusarium* multi-mycotoxin challenge compared to a control diet on antibody titers of: (**a**) Newcastle disease vaccine (NDV) and (**b**) infectious bursal disease (IBD) in broilers with or without the addition of a yeast cell wall extract (YCWE) or postbiotic yeast cell wall-based blend (PYCW) (mean ± SE). T1 = Control with no supplement and no mycotoxin challenge (CON); T2 = T1 + YCWE; T3 = T1 + PYCW; T4 = Contaminated diet (TOX: 3.0 mg/kg DON; 2.17 mg/kg 3 ADON; 104 μg/kg T 2; 79 μg/kg ZEA); T5 = T4 + YCWE; T6 = T4 + PYCW. Different superscript letters above the bars indicate significant differences between treatments, *p* ≤ 0.05 (ANOVA followed by Tukey’s multiple range test).

Responses of antibody titers against Newcastle disease vaccine (NDV) and infectious bursal disease (IBD) in the peripheral blood on day 21 and 42 are presented in Figure 1. On day 21, the NDV and the IBD antibody titers were lower (*p =* 0.039) in birds fed the *Fusarium* contaminated diet than other treatments. On day 42, the NDV titer was lower (*p =* 0.008) in birds fed the contaminated diet compared to birds fed diets containing both mitigation treatments. There was no difference among the treatments for IBD antibody titers of birds at 42 days. There was no difference in the relative internal organ weights (*p =* 0.43) on day 21 and 42, except the relative weight of the spleen on day 21, where the relative organ weight in birds fed control diet was lower (*p =* 0.006) compared to other treatments (Figure S1 in the Supplementary Section).

### 2.3. Serum Biochemistry

On day 21, the differences among treatments in total proteins, albumin measurements and the albumin-to-total protein ratio were insignificant (*p* > 0.05, Table 2). On day 42, both the total protein and albumin levels were higher in challenged birds (T4) compared to the control (T1, *p* = 0.006), but also between YCWE/PYCW under challenge conditions (T5, T6) and control (*p <* 0.005). As a consequence, the ratio of albumin-to-total protein was found to be similar among treatments (*p =* 0.384).

Blood glucose level was affected by all dietary treatments for both periods. The glucose level was lower on day 21 and 42 in all the treatment groups compared to control. On day 21, within challenged birds, YCWE- and PYCW-fed birds had lower (*p <* 0.001) glucose levels compared to birds fed the contaminated diet. However, no differences were recorded by day 42 under challenged conditions for both preventive treatments.

The mycotoxin challenge (T4) did not affect creatinine levels compared to the control (T1). In addition, there were no differences between any of the treatments in creatinine levels on day 21. However, on day 42, the creatinine level was lower (*p =* 0.001) in unchallenged birds treated with PYCW than in any other treatments.

There was no significant difference in serum uric acid levels on either day 21 (*p =* 0.211) or 42 (*p =* 0.275) across the treatments.

Cholesterol levels were not affected by the mycotoxin treatment (T4) compared to control (T1). On day 21, the cholesterol levels in PYCW supplemented groups were lower (*p <* 0.001) compared to their respective dietary control groups (control group T1 and contaminated group T4) and reached a similar concentration between PYCW treatments (T3 and T6), which amounted to an average of a 23% lower cholesterol level compared to the control.

### 2.4. Serum Enzymes: Liver Function Test

**Figure 2:**
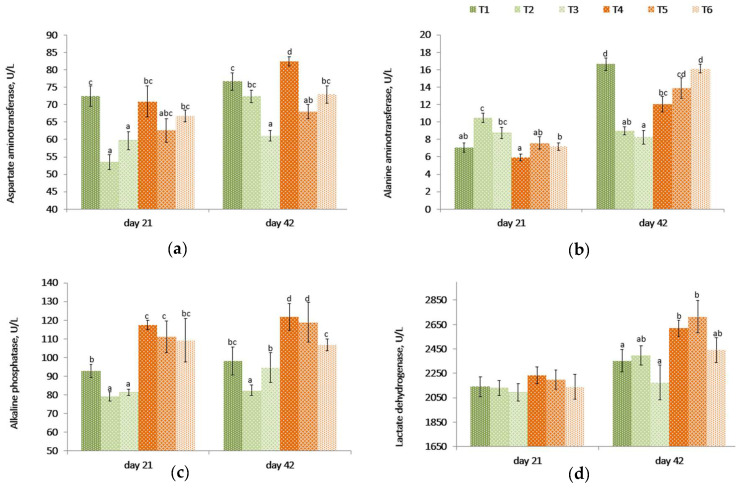
Effect of a *Fusarium* multi-mycotoxin challenge compared to a control diet fed to broilers on the circulatory levels in blood serum of four liver enzymes: (**a**) aspartate aminotransferase (AST); (**b**) alanine aminotransferase (ALT); (**c**) alkaline phosphatase (ALP); and (**d**) lactate dehydrogenase (LDH) with or without the addition of a yeast cell wall extract (YCWE) or postbiotic yeast cell wall-based blend (PYCW) (mean ± SE). T1 = Control with no supplement and no mycotoxin challenge (CON); T2 = T1 + YCWE; T3 = T1 + PYCW; T4 = Contaminated diet (TOX: 3.0 mg/kg DON; 2.17 mg/kg 3-ADON; 104 μg/kg T-2; 79 μg/kg ZEA); T5 = T4 + YCWE; T6 = T4 + PYCW. Different superscript letters above the bars indicate significant differences between treatments, *p* ≤ 0.05 (ANOVA followed by Tukey’s multiple range test).

On day 21, even though there were no differences between the challenged (T4) and unchallenged control birds (T1), the aspartate aminotransferase (AST) levels were lower (*p* < 0.001) in unchallenged birds fed PYCW and YCWE (Table 3, Figure 2) than the control group (T1). However, on day 42, birds on the contaminated diet (T4) had higher AST levels compared to other treatments. Among the unchallenged birds, the PYCW fed group had lower (T3, *p* < 0.001) AST levels. A similar effect was observed in challenged birds where lower AST levels were seen with both products compared to the contaminated diet alone (T4) after 42 days. Also, the AST blood concentrations were significantly lower in T3 supplemented birds when compared with the control group T1 following 42 days of supplementation.

The alanine aminotransferase (ALT) levels were lower for the mycotoxin challenge (T4) compared to the control (T1) at 42 days, but the difference was not significant on day 21. These levels became higher (*p <* 0.001) in birds fed YCWE or PYCW compared to their respective challenged and unchallenged dietary controls on day 21. On day 42, compared to birds on control diets (T1), the ALT levels were lower (*p <* 0.001) in birds fed YCWE and PYCW. However, in groups fed the contaminated diet, the PYCW fed group had a higher (*p =* 0.001) level of ALT compared to the control (T1), while the YCWE fed group saw only a numerically greater level of ALT. Additionally, the PYCW supplemented group (T6) had comparable ALT levels to the control (T1) receiving no mycotoxins.

The alkaline phosphatase (ALP) levels were higher (*p <* 0.005) in the contaminated diet-fed birds (T4) compared to control birds (T1). Both YCWE (T2) and PYCW (T3) fed birds had lower blood concentrations of ALP at 21 days (*p <* 0.02), and in birds fed YCWE at 42 days (*p <* 0.05). Within contaminated groups, the addition of PYCW (T6) resulted in significantly lower (*p =* 0.001) ALP levels at the end of the study compared to the challenged group (T4), and the concentrations were comparable to the control (T1).

The lactate dehydrogenase (LDH) levels were similar between treatments at 21 days (*p* = 0.813). After 42 days, the mycotoxin-challenged birds (T4) had significantly greater (*p* = 0.030) LDH levels in blood compared to the control (T1). At day 42, within the challenged and unchallenged birds, there was no difference between supplemental groups and its respective controls. Even though the recorded LDH levels for the PYCW (T6) treatment were not significantly different from the unchallenged control (T1), the LDH levels were greater in T4 and T5 when compared to the same control (T1).

### 2.5. Gastro-Intestinal pH

The pH was not affected (*p* > 0.053) by the mycotoxin challenge or from supplementation of YCWE and PYCW except in the ileum on day 42 (Table S1 in the Supplementary Section).

### 2.6. Caecal Short-Chain Fatty Acids Concentration

Table 4 and Table 5 (Figure S2 in Supplementary Section) show the proportions of short-chain fatty acids (SCFA) measured in the caeca at days 21 and 42, respectively. No significant differences were noticed for total SCFA concentration, on either day 21 or 42. However, on day 21, the cecal acetate level was higher in control (T1) fed birds compared to contaminated diet fed birds (T4). On an individual SCFA-base, acetate, isovalerate and valerate proportions significantly differed in challenged birds (T4) over the control birds (T1, *p* < 0.003) at 21 days. On day 42, only isovalerate remained different between the two latter treatments.

On day 21, butyrate was higher in PYCW supplemented birds irrespective of the diet type (*p <* 0.001). Compared within the control diet-fed birds, the butyrate level was higher in the PYCW supplemented group than in the YCWE group, nevertheless a numerical increase with the YCWE treatment could be seen compared to the control diet. Similarly, for contaminated diets, a similar trend was noticed. Isovalerate and valerate levels were significantly higher in the birds fed the contaminated diet (T4) compared to the control diet (T1). Treatments with YCWE and PYCW, regardless of diet contamination, were not different when compared to the control (T1).

On day 42 (Table 5), total SCFA and proportions of acetate, propionate, isovalerate and valerate levels were not different across treatments. Compared within birds fed non-contaminated diets, the iso-butyrate level of PYCW treatment (T3) was higher (*p =* 0.05) than its control (T1). Additionally, the butyrate level in the birds fed a contaminated diet supplemented with PYCW (T6) was higher (*p <* 0.001) compared to the rest of the treatment groups, with the exception of challenged birds fed YCWE (T5).

### 2.7. Intestinal Histomorphology: Gut Health

**Figure 3:**
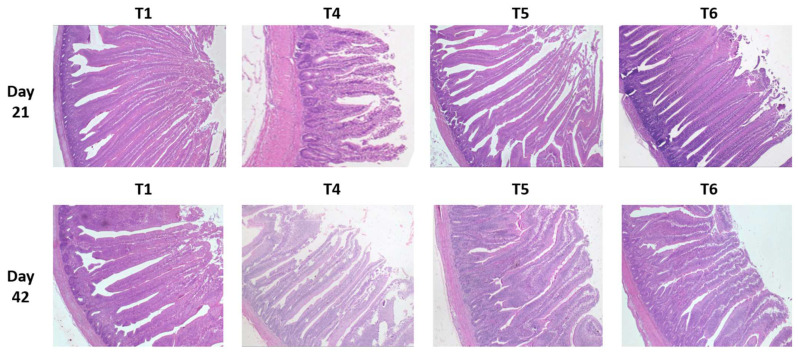
Histological observations of intestinal villi using light microscopy after staining of duodenum intestine tissue samples collected on day 21 and 42 for broilers fed *Fusarium* multi-mycotoxin challenge compared to a control diet with or without the addition of a yeast cell wall extract (YCWE) or postbiotic yeast cell wall-based blend (PYCW). T1 = Control with no supplement and no mycotoxin challenge (CON); T4 = Contaminated diet (TOX: 3.0 mg/kg DON; 2.17 mg/kg 3-ADON; 104 μg/kg T-2; 79 μg/kg ZEA); T5 = T4 + YCWE; T6 = T4 + PYCW.

The histo-morphological measurements of the three compartments of the small intestine are reported in Table 6 and their microscopic observations of the duodenum on day 21 and day 42 are shown in Figure 3 (Figures S3 and S4 in the Supplementary Section).

The significant impact of mycotoxins (T4) could be seen at 21 and 42 days in all digestive compartments for villus height (VH) and crypt depths (CD) compared to the control birds, (T1) while the crypt depth ratio (VH/CD) remained significantly different only at 21 days.

When evaluating the effect of tested supplements, generally, at both time periods, tVH of birds fed either supplement was greater (*p <* 0.005) in all three segments (duodenum, jejunum, and ileum) compared to their respective controls (T1 and T4). PYCW (T6) had significantly greater VH when compared to the control (T1), while the mycotoxin challenge (T4) was significantly shorter. Except for jejunum and ileum at 42 days, YCWE, exhibited the same metrics as PYCW.

On day 21, within the unchallenged treatments, only birds fed YCWE (T2) had shorter CD in the duodenum (*p <* 0.05) and jejunum (*p <* 0.001) compared to the control group, whereas PYCW (T3) did not exhibit any difference. However, in birds fed the contaminated diet, CD was greater (*p <* 0.001) in birds supplemented with YCWE (T5) compared to its control (T4). Additionally, on day 42, within the mycotoxin-challenged birds, CD of the supplemented YCWE and PYCW diets were greater (*p <* 0.001) than their control (T4). Both tested materials had similar CD measurements compared to the T1 treatment after 42 days.

Considering the VH/CD ratio of the unchallenged birds, values for YCWE and PYCW were greater (*p <* 0.05) in all intestinal tissues at 21 days, but no differences were observed on day 42. For the mycotoxin-fed birds, VH/CD for the duodenum and jejunum of the PYCW treatment (T6) had significantly higher ratios (*p <* 0.001) compared to the contaminated diet group (T4) on day 21. Whereas after 42 days, both PYCW and YCWE exhibited lower VH/CD values compared to the contaminated diet (T4), and the values were similar to the control (T1).

### 2.8. Goblet Cell Count

On day 21, the relative concentration of goblet cells in the intestinal villi of birds receiving the contaminated diet was lower (*p* = 0.002) in the duodenum compared to the control treatment (T1) but was not different in other intestinal tissues.

**Figure 4:**
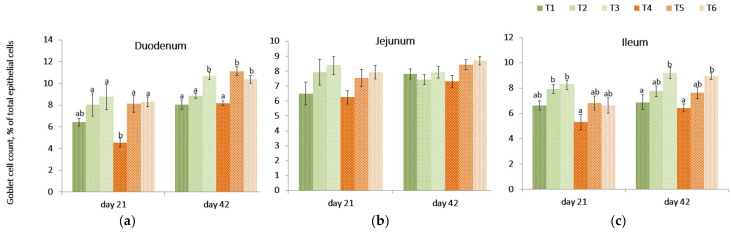
Effect of a *Fusarium* multi-mycotoxin challenge compared to a control diet on goblet cell count (expressed as a percentage of total epithelial cells) in three sections of the small intestinal tissues: (**a**) the duodenum, (**b**) the jejunum, (**c**) and the ileum of broilers with or without the addition of a yeast cell wall extract (YCWE) or postbiotic yeast cell wall-based blend (PYCW) (mean ± SE). T1 = Control with no supplement and no mycotoxin challenge (CON); T2 = T1 + YCWE; T3 = T1 + PYCW; T4 = Contaminated diet (TOX: 3.0 mg/kg DON; 2.17 mg/kg 3ADON; 104 μg/kg T-2; 79 μg/kg ZEA); T5 = T4 + YCWE; T6 = T4 + PYCW. Different superscript letters above the bars indicate significant differences between treatments, *p* ≤ 0.05 (ANOVA followed by Tukey’s multiple range test).

The PYCW and YCWE supplementation evaluated at 21 day in the duodenum remained unchanged when compared to the control (T1) but was significantly different compared to the mycotoxin challenge (T4). (Figure 4, Table S2 in Supplementary Section). A similar trend was observed in the jejunum, but no significant differences were found across diets (*p* = 0.123). The goblet cell count in the ileum was lower (*p* = 0.001) in birds fed a contaminated diet compared to the control diet containing either supplement (T2, T3). On contrast, within contaminated diet groups, the goblet cell count on day 21 for ileal tissue was higher in YCWE fed birds (*p* = 0.033) and tended to be higher (*p* = 0.061) in the PYCW supplemented group when compared to challenged birds (T4).

On day 42, the PYCW fed birds had a significantly higher goblet cell concentration count in duodenal and ileal tissues compared to control (T1) and contaminated diet-fed birds (T4). Additionally, there was a tendency (*p* = 0.052) for higher goblet cell concentration counts in the ileum when birds were supplemented with YCWE. In duodenal tissues, goblet cell concentration counts were highly significant for both products in mycotoxin-challenged birds (*p* < 0.001). Overall, at both time points, YCWE and PYCW resulted in higher goblet cell counts above control values, and this trend was significant for contaminated diets.

### 2.9. Production Efficiency European Production Efficiency Value (EPEF)

Upon computation of EPEF values, it was noted that birds fed the contaminated diet (T4) had a lower EPEF value (*p* = 0.001) than the control fed birds (T1). (Table 7). Supplementation with YCWE or PYCW (T5, T6) compared to the contaminated diet (T4) at the inclusion rate of 0.2% produce greater EPEF values. Furthermore, the birds receiving both tested yeast-based products did not differ from the control fed birds (T1, *p* > 0.20).

## 3. Discussion

Natural *Fusarium* toxins contamination of feedstuffs represent an important unavoidable economic issue to animal production and ultimately to broiler health. Along with the implementation of critical control steps to prevent mycotoxin contamination, supplementation of certain feed ingredients that could reduce the adverse effects of mycotoxins in the animals are used as a complementary step to a successful mitigation program [19,45,46,47,48]. Yeast cell wall extracts have been shown to mitigate the toxic effects of DON poultry [49,50]. To further enhance the mitigation effects of YCWE, especially in handling a plurality of mycotoxins present in naturally contaminated diets, a proprietary blend of post-biotic functional bioactive constituents in addition to the yeast cell wall was formulated (PYCW). This product was thus designed to reduce the mycotoxin impact by not only limiting the bioavailability and toxin absorption, but also by indirectly competing mitigating with the harmful effects of mycotoxins within the animal organism. Recent research has reported the benefits of PYCW preparations and was proposed as a possible tool for reducing the negative effects of mycotoxicosis induced by *Fusarium* toxins in swine [45]. Therefore, the present research is bringing novel insight into the use of PYCW applied to broiler chickens under commercial production conditions, at an inclusion rate of 2.0 kg/T in the diet and evaluation of its mitigation properties toward naturally occurring *Fusarium* toxins, in comparison to the original YCWE, used at the same inclusion rate.

As intended in the current study, T-2 toxin and DON levels were in the median range of concentrations found in field surveys [51]. The presence of one of the DON metabolites, 3-ADON, could enhance the overall effects of DON [52,53]. While DON has low toxicity among mycotoxins, its anorexic and emetic potencies can play a detrimental role in animal production, often greater than other potent trichothecenes, such as T-2-toxin [54]. Similarly, comparable to DON-3-glucoside, 3-ADON and 15-ADON can be converted to DON in the digestive environment due to enzymatic activities, and thus contribute to the overall DON negative effects [55]. Our study demonstrated that the presence of naturally contaminated dietary *Fusarium* toxins adversely affected the health and production performance of broiler birds. The inclusion of PYCW and YCWE in the diet at 2.0 kg/T was able to significantly mitigate those effects. The main impact of *Fusarium* toxins was observed on the gastrointestinal tract, immune system and liver functional traits.

A higher feed intake (FI) during the grower phase and reduced body weight (BW) in birds challenged with *Fusarium* toxins was evidenced when compared to a non-contaminated diet. Additionally, a better FCR in the final stage of the study was observed in birds fed PYCW and YCWE, which corroborates other findings highlighting the effects of DON specifically during the later stages of growth [56], indicating an age-related difference in uptake, metabolism and excretion. However, the zootechnical impact of DON on performance traits varied between studies, depending on the concentration and source of toxin-natural contamination versus synthetic mycotoxins—[3,57] and duration of exposure to the challenge [58]. Differences in biotic and abiotic environmental factors not necessarily captured during those trials could also contribute to the variation in reported results. In our study, the presence of naturally contaminated *Fusarium* toxins caused lower productive performance and suggested effects that could be beyond the simply additive effects of co-contaminants. The chronic intake of naturally contaminated feedstuffs with multiple *Fusarium* mycotoxins throughout the bird’s life may also contribute to a loss in productive performance [59]. In addition, BW reduction was also characterized in broiler chicks consuming a contaminated diet containing 1.7–12.2 mg/kg DON [60], suggesting a major impact on highly productive birds in commercial settings [61].

The presence of PYCW at 0.2% (*w/w*) in the diet resulted in a significantly higher BW of birds compared to the challenged treatment (TOX) or to birds fed a control diet. Additionally, at an identical inclusion rate, the performance of birds fed PYCW was greater compared to birds fed YCWE. Although mycotoxins in the diet did not affect the FI, the FCR was higher in birds fed the contaminated diet. At the end of the 42-day study period, challenged broilers fed PYCW and YCWE showed lower FCR. Results from various studies have suggested that the FCR can be affected by several factors including the type of mycotoxin, its concentration, but also by the type of mitigation product tested and its inclusion rate. In one study, 0.05 or 0.1 kg/T YCW supplemented diet in multi-mycotoxin challenged birds did not improve dietary FCR after 28 and 42 days [18], but the same product at 0.05% provided lower FCR after 35 days of consuming a diet naturally contaminated with lower to moderate levels of mycotoxins, composed mainly of AFB1 [62]. Weaver et al. [59] examined the impact of naturally contaminated feed with *Fusarium* toxins containing mostly DON (1.2 to 2.0 mg/kg of feed), and to a lesser extent fumonisins (2.5 to 5.0 mg/kg). A significant lower FCR was observed with the addition of 0.2 kg/T of YCWE in the diet of poultry broilers compared to the one fed the mycotoxin containing challenge diet alone.

Along with performance, the immune response was also affected by the multi-mycotoxin challenge in this study. However, the lymphoid organ weight relative to final body weight either at day 21 or 42 was minimally impacted by the mycotoxin challenge or PYCW/YCWE supplementation. Under similar experimental conditions, contradictory results have been observed where feeding 16 mg DON/kg of feed for 21 days did not change the relative weight of bursa of Fabricius [63,64], but decreases were seen in other studies where a *Fusarium moniliforme* corn culture spiked multi-contaminated diet were fed at 0.5, 5, or 25% [12]. Only a few studies have reported an increase in the relative organ weight of the spleen in broiler birds fed low levels (1.68 mg/kg) [60] to high levels (5 mg/kg) of DON, which could be due to cellular proliferation, DNA damage at the cellular level or irritation of the immune organ [65]. Eshak et al. [66] reported that bursa weighed more in chickens treated with sodium butyrate, which was explained by an increased thickness of the parenchymal areas [66,67,68]. However, many other studies have indicated that the relative organ weight of the spleen was not affected by DON fed between 5.9 and 18 mg/kg to broilers [14,69]. Even though immune organ weights weren’t dramatically affected, the vaccinal immune response was consistently lower under the contaminated treatment condition above 5 mg/kg of DON in broilers [70]. The low NDV and IBV titers under the tested challenge would indicate that low levels of mycotoxins could reduce the immune response, potentiated by a co-mycotoxin exposure and a long duration of exposure (21 vs. 42 days). Several studies have reported lower NDV titers at 28 and 42 days of age in broilers exposed to a mixture of *Fusarium* mycotoxins [71]: in White Leghorn chicks fed 18 mg of DON/kg of feed for 18 weeks [69]; in broilers fed 12.2 mg of DON/kg of feed for 5 weeks [60]. This was mainly linked to protein synthesis inhibition [64]. Concerning IBV, contradictory results have been obtained where antibody titers were depleted in broiler chickens receiving a high level DON (10 to 12.6 mg/kg for 5 weeks) [72], but no effects were observed at lower levels of DON (4.5 to 10 mg/kg of feed for 35 days) [39] or similar levels (12.2 mg/kg for 28 days) [60]. Our results indicated that the *Fusarium* toxins could modulate the immune response and antibody titers against NDV, which was counteracted by the addition of PYCW.

Among blood parameters, glucose and cholesterol were significantly higher on day 21 in birds fed the mycotoxin challenge diet, indicating some level of hepatotoxicity. On day 42, the total protein and albumin levels were higher in birds fed the contaminated diet compared to the non-contaminated diet. According to previous research, the impact of mycotoxins on blood metabolites of broilers can differ. Dietary DON at 3.0 mg/kg fed to broilers over 42 days did not impact blood metabolites [73], but cholesterol and triglyceride levels increased when administered at 10 mg/kg fed for 35 days [74]. Conversely, a meta-analysis has shown decreased cholesterol and triglycerides levels in broilers exposed to DON, T-2, OTA, and ZEA, suggesting a shift of triglyceride and cholesterol from the blood to the liver [72,75,76,77,78,79]. Additionally, ZEA and T-2 were found to affect blood metabolites at 21 days but failed to induce any changes at 35 days [62]. Therefore, it can be inferred that *Fusarium* mycotoxins present in combinations could alter the liver metabolism leading to changes in circulation and storage of certain blood metabolites. The supplementation of PYCW resulted in levels equivalent to those of the control. Generally, YCW-based supplements have been shown to influence growth when supplemented to birds exposed to low background levels of toxins in the diet, suggesting more efficient lipid uptake [80]. In the present study, serum cholesterol was lower in the supplemented groups, as found by Ghahri et al. [81], reflecting possible hepato-protective properties and sparing effects of PYCW. This could be associated with altered storage maintenance and lower cholesterol release into the systemic circulation. Similarly, an earlier study showed that YCW supplemented to birds challenged with mycotoxins could preserve the toxin-induced breakdown of glycogen (hyperglycemia) [82].

Furthermore, supplementing PYCW or YCWE, irrespective of dietary toxin levels, did not affect blood protein levels or the uric acid levels, in contrast to observations from a previous study (data unpublished), where PYCW added to an uncontaminated diet resulted in higher albumin/total protein indicating mediation of protein synthesis in the liver. These observations suggest the participation of butyric acid, which is present in PYCW, in energy homeostasis and regulation of sugar absorption or improvement of mitochondrial function. Butyrate could act as an effector in several types of intestinal and liver cells that mediate protein synthesis, and could cause a slight relapse depression in albumin production, thus appearing as a decrease in the albumin/total protein ratio [83]. Lower serum protein also reflects the ability of birds to mobilize and utilize the protein even after the body has reached its tissue holding capacity. Conversely, during mycotoxicosis, serum total protein and albumin concentrations in chickens may be reduced due to the inhibition of amino acid transport and mRNA transcription, and the potential direct interaction of DON with ribosomal units, leading to the inhibition of protein synthesis [84,85]. YCW based supplementation (at 0.05–0.10% to the diet, *w/w*) led to significantly higher serum protein levels when compared to the toxin-alone fed group [38,62]. Similarly, YCW at 0.10 % was successful at significantly increasing the blood protein in broilers during aflatoxicosis [61,82]. Additionally, the presence of vitamin C in PYCW could also have contributed towards higher performances of the birds as noticed in other studies. Generally, vitamin C has been shown to oxidize fatty acids yielding energy and involved in calcium and vitamin D3 metabolism [86,87] and thus participating in energy homeostasis, the regulation of sugar absorption and the lowering of blood cholesterol levels. In addition, improved mitochondrial function, decreased ROS levels, and participation in protein synthesis in intestinal and liver cells has been observed [88].

Generally, an increase in blood enzymatic activities is an indication of hepatic disorders, chronic liver damage, hepatic leakage, obstruction of the biliary duct, or kidney problems [39,74,89]. With the higher blood levels in liver integrity enzymatic biomarkers (AST and ALP on day 21 and 42 and LDH at 42 days), our results were in agreement with previous reports of higher AST level in chickens fed DON at a high dose (15 mg/kg of feed) [90], but also with elevated ALP and AST in chicken fed DON at much lower concentration, 2.95 mg/kg of feed [65,91]. Regarding ALT, opposing reports have been produced, showing either elevated levels in broilers exposed to 3.0 mg DON/kg feed for six weeks [92], or lowered ALT levels in broilers fed DON at 10 mg/kg for 35 days [74]. When challenged birds were supplement with PYCW, blood enzyme (ALT and ALP) levels were lower and comparable to the control, indicating potential mitigation of the liver toxicity issues observed with the mycotoxins diet.

Previous research has shown that mycotoxin contamination can alter the enterocyte integrity in poultry and can result in poor nutrient utilization, which in turn may alter long-term health and performance. In our study, the pH of the gut did not change with the mycotoxin challenge. However, the intestinal VH and CD were lower throughout the small intestine in birds fed a contaminated diet. Such aberrations can compromise key functions of the gastro-intestinal tract, including the decreased surface area available for nutrient absorption, modulation of nutrient transporters, or loss of barrier function as indicated in other studies with *Fusarium* toxins [60,93,94,95]. Similar results in broiler experiments were found, where DON concentrations of 3 mg/kg significantly decreased VH and transepithelial electrical resistance in the duodenal segment [96]. DON fed for 34 days at the concentration of 7.9 mg/kg decreased VH (506 vs. 680 µm) and reduced CD (85 vs. 115 µm) in jejunal and ileal tissues [56]. Concentrations of 7.54 mg DON/kg of feed fed for 21 days were also reported to decrease VH and CD in the duodenum and jejunum [97]. Santos and van Eerden [57] demonstrated a similar impact in broiler birds fed moderate levels of the synthetic form of DON (2.3 mg/kg of feed), which became more severe when the same proportion of DON originated from a natural source, as seen in other studies [93,94,98]. *Fusarium* toxins including DON are implicated in triggering epithelial cell proliferation or inhibition of epithelial cell differentiation and apoptosis leading to shortening of VH [99], consequently impairing apparent nutrient digestion [100]. The changes in the CD represent a compensatory mechanism to apoptosis during the toxic assault in an attempt to regenerate enterocytes that progress along the villus towards the tip [101]. The intestinal structural changes can be assessed by calculating the VH/CD ratio where the reduced rate of villi height growth with respect to crypt depth regeneration infer abnormal architecture, ultimately affecting nutrient absorption and gut barrier functions [101]. In our study, the lower VH/CD ratio observed for the challenged diets could be attributed once more to inhibition of protein biosynthesis, or cell differentiation or apoptosis induced by the dietary toxins, as observed in other studies [97,102]. Mechanistically, changes in the gene expression of important markers have been reported, such as the villin protein composing the brush border (VIL1) or the protein receptors GLUT1 or PEPT-1 [3]. This latter protein is a key receptor in the absorption of important macronutrients, such as amino acids. The lack of gut health aberrations in YCWE and PYCW supplemented birds suggest a beneficial mitigation role. This has been previously observed for YCW, used at 2.0 kg/T in the diet containing *Fusarium* mycotoxin over 21 days in broilers consuming 2.0 or 4.0 kg of YCWE/T of feed during a mycotoxin challenge [59]. In addition to the YCW fraction, butyrate in PYCW can also act as a source of energy for the enterocytes [103], and could affect cell mitosis in the crypts and further protect the mucosal epithelium from injury, alleviating the enterohepatic stress [104] and sustaining the intestinal absorptive area by promoting villus growth [105,106].

The goblet cell concentration count in the duodenum and ileum was lower in birds exposed to the *Fusarium* contaminated diet. The goblet cells have a key gate-keeping role directly involved in mucin production forming the glycocalyx coat over the epithelial layer of the intestine that acts as a selective barrier for entero-toxins and bacterial assault, while enabling the transfer of important nutrients, micro- and macro-elements [49]. The mucous along with the Paneth cell secretions also produces an antibacterial gradient that protects the animal from intestinal pathogenic populations [107]. Earlier, a study has demonstrated lower goblet cell count concentrations in duodenum during a *Fusarium* mycotoxins challenge, mainly with DON at 2.26 mg/kg feed in broilers, [59]. DON genotoxicity specifically targets cells responsible for high protein turnover and activates cells, thus causing changes in gut morphologies and leading to a compromised gut barrier [108,109]. The greater number of goblet cell concentrations in the duodenum and ileum with PYCW supplementation indicate that PYCW may uphold the goblet cells activities by regulating its mucin gene and could positively contribute to the protective mechanisms in the gut as demonstrated [110]. This could also protect the animal from potential opportunistic pathogens, which could further exacerbate the impact of the mycotoxin.

The analysis of SCFA in the hind gut revealed a lower level of butyrate in birds fed a contaminated diet and higher levels of butyrate in birds supplemented with PYCW. Short-chain fatty acids play an important role in maintaining the structural and functional integrity of intestinal epithelial cells [40,111]. The health benefits from PYCW can be attributed to several of its components, including butyrate, vitamin C and essential oils, in addition to the yeast cell wall carbohydrate fraction, as in the YCWE treatment itself, having demonstrated mitigation properties [14,58]. With regard to gut health, few studies are available in the literature that disclose the benefits of butyrate in broiler chickens [112,113,114]. To some extent, SCFA can control bacterial infections and act as a potential antibiotic replacement [115,116]. They may also improve protein and energy digestibility by reducing microbial competition for nutrients with the host, [117] or even reduce subclinical infections [118,119], where butyrate is considered to have the most bactericidal property against acid-intolerant species such as *E. coli* and *Salmonella* spp. [120]. A negative correlation between the concentration of SCFA and the concentration of *Enterobacteriaceae* has been observed in the ceca of broilers [121]. Additionally, *Bacteroidetes* (gram-negative) and *Firmicutes* (gram-positive) are most abundant in the intestine, where the former are known to produce acetate and propionate and the latter to produce butyrate [122]. *Bacteroidetes* have been linked to a decrease in nutrient absorption while *Firmicutes* have been linked to increased nutrient absorption [123]. This could support the argument that the SCFA induced by PYCW promoted better nutrient absorption from the gut. This change in population, significantly for *Bacteroidetes*, was also observed when YCWE was used in conjunction with a mycotoxin challenge in young pigs [27],. and was also reflected in one of our preliminary studies (unpublished), which indicates the involvement of the yeast cell wall constituents in this shift in population.

The standard calculation of EPEF values has been used to determine production efficiency of broilers production, which is determined from the broiler performance parameters including livability, BW and FCR [124]. The overall EFEP values were found to be in the lower range compared to studies performed under similar conditions and for the same genetic breed [59]. Those dissimilarities could be explained by lower initial BW in our study, but with higher growth rates. In addition, quality of feed, feed nutrient density, and external stress factors including ambient temperature (which averaged 26.6 °C in our study) are also to be considered as potential factors influencing animal responses. In our study, higher EPEF (*p* ≤ 0.004) was recorded for birds supplemented with both YCWE and PYCW compared to birds fed the contaminated diet, and there was no difference between the supplemented and CON group. This indicated more consistent and uniform body weights in birds between YCWE or PYCW supplemented birds and CON. This also indicates that supplemented birds had a better health status during mycotoxin exposure, allowing more efficient performance, as observed previously [59,125,126].

## 4. Conclusions

Overall, diets supplemented with both YCWE and PYCW at an inclusion of 0.2 kg/T in the diet were supportive of animal health and, as indicated by the integrity of the mucosal morphology of the small intestine, while supporting greater goblet cell concentrations, improved liver function and higher butyrate levels in the hindgut, supporting better productivity. We determined that the control diet fortified with PYCW had additional advantages arising from its compositional nutritional attributes, which translated into beneficial effects on some plasma parameters and gut physiology. Thus, the strategic formulation of PYCW as an extension of YCWE could be applied to the diet of broiler chickens to optimize performance when birds are exposed to a naturally contaminated *Fusarium* mycotoxin challenge.

We found that the combination of *Fusarium* toxins at levels under the regulatory maximum recommended levels in the feed can still adversely affect a bird’s production performance, immunity, liver function and gut health. The nutritional attributes of PYCW, and to a lower extent YCWE, helped mitigate the mycotoxin impact throughout the experimental period at the inclusion rate of 0.2% (*w/w*) in the diet. Birds supplemented with PYCW or YCWE were able to maintain villi architecture and goblet cell count, preserve liver function and thus sustain animal productive performances at normal rates, as seen with EPEF calculations. The YCWE also presented beneficial effects which were noticeable during the starter and grower phase of the birds.

## 5. Materials and Methods

### 5.1. Experimental Design and Diets

A total of 720 one-day-old non-vaccinated male Cobb broiler chicks were obtained from a commercial hatchery and kept in a floor pen with wood shavings as a litter. The ambient temperature was kept at 32 °C for the first week and gradually lowered by 3 °C the second week and maintained at room temperature (Average of 26.6 °C) thereafter. At the beginning of the experiment, the birds were individually weighed, wing banded and distributed into six treatments (120 birds/diet, 8 pens/diet, 15 birds/pen) according to a completely randomized experimental design. Treatments included T1: control non-contaminated diet (CON); T2: unchallenged treatment comprised of T1 + YCWE; T3: unchallenged treatment comprised of T1 + PYCW; T4: Mycotoxin challenge treatment; T5: T4 + YCWE; and T6: T4 + PYCW. Both test articles-YCWE and PYCW-were added in place of corn at an inclusion rate of 2.0 kg/T to the control diet to prepare the experimental diet. Iso-nutritive feeds, based on corn and soybean meal, without animal by-products and with the inclusion of anticoccidials and antibiotics, were formulated according to the standards used in commercial farms. The feed formulation supplied the nutritional requirements determined by the Nutrient Requirement Council [127] following a three-phase (starter (1–14 days), grower (15–28 days) and finisher (29–42 days)) formulation (Table 8). Birds were vaccinated, according to the local schedule, against Marek’s disease on day one (S/C route), New Castles disease (ND) on day seven and infectious bursal disease (IBD) on day 14 (I/O routes). Booster doses for ND and IBD were given on day 21 and 28, respectively. The birds were fed *ad libitum* throughout the experimental period. Products tested included a post-biotic yeast cell wall-based blend (PYCW, Mycosorb D+™) and a yeast cell wall extract (YCWE, Mycosorb A+^®^), both procured from Alltech Inc., Nicholasville, KY, USA. The PYCW is a proprietary blend of postbiotic functional bioactive constituents containing the yeast cell wall of *Saccharomyces cerevisiae*, organic acids (*n*-butyric acid), vitamins (ascorbic acid), and essential oils (rosemary extract), while YCWE was mainly composed of yeast cell wall extract and an algal source.

### 5.2. Contaminated Diet Preparation

The *Fusarium* species were cultured on oatmeal agar (25 °C, 14 d), and the conidial suspension was separated by centrifugation. The toxins were subsequently produced by solid-state fermentation on 50-g autoclaved-sterilized grain (*n* = 100; 35% moisture, 22 °C) inoculated with a 1-mL suspension containing 10^5^ of fungus spores (*F. culmorum* ITCC148 on broken maize/rice; *F. sporotrichoides* ITCC1894 on wheat). The samples were autoclaved at 121 °C for 15 min and oven-dried before LC-MS/MS analysis for mycotoxins [128] (37+™ Laboratory, Alltech Bioscience Center, Dunboyne, Ireland). The analyzed toxin concentration in the fermented media was used to calculate the final inclusion rate to obtain the desired concentration of mycotoxins in the diet (DON 3.0 mg/kg and T-2 104 μg/kg). 1.55 kg of *F. sporotrichoides* inoculated wheat media was used to obtain 104 μg/kg T-2 and 1.25 kg of *F. culmorum* inoculated rice media was used to obtain 3.0 mg/kg DON in the treatment diet. The initial concentration of the *Fusarium* toxins in two batches of fermented media was quantified by LC-MS/MS and the calculated final concentration of toxins in the treatment diet are shown in Table 9.

### 5.3. Sampling and Analysis—Growth Performance and Organ Weights

Birds were weighed individually on a weekly basis, feed consumption was recorded weekly, and mortality was recorded as it occurred. The body weight (BW) and feed intake (FI) were measured weekly and the feed conversion ratio (FCR) was calculated. On day 21 and 42, two birds/pen (16 birds/treatment) were randomly selected, weighed and sacrificed by cervical dislocation for the collection of internal organs. For internal organs assessment, immediately after the chickens were killed by exsanguination (*n* = 16), all thymic lobes, spleen and bursa of Fabricius were separated, trimmed of the adipose tissue, and weighed to determine the relative organ weights.

### 5.4. Blood Parameters—Serum Metabolites, Liver Function Test and Immunological Assessment

At day 21 and 42, two birds per replicate (*n* = 16) were randomly selected and weighed. Before killing, 5 mL of blood was collected from the brachial vein using serum collection tubes (23-gauge needle; Becton Dickinson Vacutainer Systems, Franklin Lakes, NJ, USA), for analysis of serum metabolite (albumin, total protein, glucose, cholesterol, uric acid, creatinine), liver enzymes (Alkaline Phosphatase (ALP), Alanine Aminotransferase (SGPT or ALT), Aspartate Aminotransferase (SGOT or AST) and Lactate Dehydrogenase (LDH)) and to perform immunological assessment (NDV and IBD titers) according to the procedure recommended by commercial analytical kits manufacturer (Sigma-Aldrich, Saint Louis, MO, USA). Immediately after the collection of blood, serum was separated by gravimetry (30 min on ice) and samples were centrifuged (3000× *g* at 4 °C for 15 min), aliquoted (1.2 mL) in 1.5 mL centrifuge tubes (Fisherbrand, Fisher Scientific, Hampton, NH, USA) and stored at −24 °C in a freezer (Thermo Fisher Scientific, Waltham, MA, USA) for further analysis. The immune responsiveness was assessed in terms of humoral immune responses, mainly for antibody production to NDV and IBD vaccines. The antibody titer was calculated as per the equations provided by the manufacturer.

### 5.5. Measurement of Gastro-Intestinal pH and Caecal SCFA Concentration

The gastrointestinal tracts were removed and the content from the duodenum, jejunum and ileum were collected for measurement of gastrointestinal pH and the cecal content was collected for SCFA quantification. For the pH, the sample (0.6 g) was suspended in 2.4 mL distilled water and shaken vigorously before the measurement [129]. For SCFA analysis, the cecal contents were immediately placed into sterile tubes on ice and transferred to the laboratory. After thawing at room temperature, the concentrations of SCFA of each digesta sample from the caeca were measured using gas chromatography (Agilent Technologies, 7890B, Santa Clara, CA, USA with Autosampler PAL RSI 85, CTC analytics, Switzerland) according to the method described by Jensen and Gerdes [130].

### 5.6. Gut Histomorphology and Goblet Cell Count

Intestinal tissue samples (*n* = 16) were collected from the duodenum (midpoint), jejunum (midway between the bile duct and Meckel’s diverticulum) and ileum (near the ileocecal junction). The samples were flushed with buffered saline and fixed in 10% neutral buffered formalin for 2 days. Samples were embedded in paraffin wax after dehydration in ethanol and clearing using xylene. The embedded tissue samples were sectioned at 5 μm using a microtome (Microm GmbH, Walldorf, Germany) and stained with hematoxylin and eosin. Sample sections were captured at 10× magnification under a microscope (Olympus, Olympus Corporation, Tokyo, Japan) and morphometric indices were determined as described by Iji et al. [131]. Intestinal tissue samples collected from 3 intestinal segments-ileum, duodenum and jejunum (*n* = 16 per treatment)-were measured for villus height, crypt depth and goblet cell count in 15 areas that were well oriented vertically.

### 5.7. Effciency Calculation

The European Production Efficiency Factor (EPEF) was assessed by using the performance parameters measured in the study and calculating the efficiency of broilers fed the experimental diets. The following formula was used for the calculation as described by Marcu et al., 2013 [124]:

(1) $$\mathrm{EPEF}\;=\frac{\mathrm{Viability}\times \mathrm{BW}}{\mathrm{Age}\times \mathrm{FCR}}\times 100$$

where the viability is expressed in %; BW is the animal average body weight in kg; the age in days; and FCR the feed conversion ratio expressed in kg feed consumed by kg of body weight.

### 5.8. Statistical Analysis

Data were analyzed by one-way analysis of variance (ANOVA) using SPSS statistical software (Ver. 16.0 for windows, SPSS Inc., Chicago, IL, USA). Differences among treatments were examined using Tukey’s multiple range test according to the model yij = µ + ai + eij; where yij = jth observation subjected to the treatment i; µ = overall mean; ai = effect of treatment; eij = residual error. Hypothesis-driven planned contrast comparisons for specific treatment group means was performed: T1 vs. T2 and T3 to evaluate the effects of YCWE and PYCW in the absence of a challenge; T4 vs. T5 and T6 to evaluate the effects of YCWE and PYCW in the presence of a challenge; T1 vs. T4 to evaluate the effects of the challenge; using the difference of least square means at the *p* < 0.05 level, if a significant main effect was obtained from the ANOVA. The Bonferroni-type, based on a student’s *t* test distribution, was utilized for paired contrasts. Prior to analysis, data was evaluated for homogeneity and normality using the Levene’s and Shapiro’s test, respectively. For antibody titer values, the data was transformed to log values before analysis. Differences were considered significant when *p* *<* 0.05 and *p*-values between 0.05 and 0.10 were considered as a tendency in differences. Data are presented as means and their pooled standard errors.

## Figures and Tables

**Table 1:** Comparison of the weekly performances (body weight, feed intake, feed conversion ratio) of broilers fed a *Fusarium* contaminated diet (TOX) compared to a control diet (CON), with or without supplementation of a yeast cell wall extract (YCWE) or a postbiotic yeast cell wall-based blend (PYCW) added at an inclusion rate of 2.0 kg/T (mean ± SE, *n* = 120).

	Days					
Treatments *	7	14	21	28	35	42
**Body Weight (BW), kg**						
T1: CON	0.138 ± 0.001 ^ab^	0.313 ± 0.003	0.633 ± 0.007 ^ab^	1.062 ± 0.013 ^a^	1.595 ± 0.022 ^bc^	2.145 ± 0.024 ^b^
T2: CON + YCWE	0.142 ± 0.001 ^ab^	0.319 ± 0.003	0.659 ± 0.007 ^b^	1.061 ± 0.013 ^a^	1.628 ± 0.018 ^c^	2.137 ± 0.021 ^b^
T3: CON + PYCW	0.144 ± 0.001 ^b^	0.326 ± 0.004	0.66 ± 0.008 ^b^	1.126 ± 0.016 ^b^	1.65 ± 0.023 ^c^	2.257 ± 0.03 ^c^
T4: TOX	0.139 ± 0.001 ^ab^	0.315 ± 0.003	0.627 ± 0.006 ^a^	1.069 ± 0.015 ^ab^	1.505 ± 0.018 ^a^	1.931 ± 0.019 ^a^
T5: TOX + YCWE	0.136 ± 0.001 ^a^	0.319 ± 0.003	0.644 ± 0.006 ^ab^	1.076 ± 0.014 ^ab^	1.521 ± 0.011 ^ab^	2.178 ± 0.021 ^bc^
T6: TOX + PYCW	0.136 ± 0.001 ^a^	0.32 ± 0.003	0.647 ± 0.007 ^ab^	1.09 ± 0.014 ^ab^	1.58 ± 0.023 ^abc^	2.122 ± 0.033 ^c^
Main effect, *p*-Values	0.002	0.167	0.006	0.018	<0.001	<0.001
Contrast, *p*-Values:						
T1 vs. T2	0.145	0.244	0.015	0.981	0.266	0.798
T1 vs. T3	0.013	0.015	0.020	0.003	0.097	0.004
T1 vs. T4	0.706	0.625	0.543	0.735	0.002	<0.001
T4 vs. T5	0.214	0.476	0.073	0.738	0.450	<0.001
T4 vs. T6	0.213	0.292	0.039	0.337	0.014	<0.001
**Feed Intake (FI), kg**						
T1: CON	0.123 ± 0.001	0.482 ± 0.004	1.110 ± 0.010 ^b^	1.778 ± 0.022	2.909 ± 0.051	4.537 ± 0.05
T2: CON + YCWE	0.125 ± 0.001	0.469 ± 0.005	1.050 ± 0.012 ^a^	1.726 ± 0.015	2.805 ± 0.022	4.321 ± 0.047
T3: CON + PYCW	0.123 ± 0.001	0.467 ± 0.005	1.035 ± 0.008 ^a^	1.773 ± 0.021	2.857 ± 0.036	4.551 ± 0.147
T4: TOX	0.124 ± 0.001	0.474 ± 0.001	1.045 ± 0.013 ^a^	1.790 ± 0.018	2.858 ± 0.078	4.493 ± 0.094
T5: TOX + YCWE	0.123 ± 0.002	0.485 ± 0.016	1.046 ± 0.013 ^a^	1.729 ± 0.035	2.751 ± 0.054	4.412 ± 0.063
T6: TOX + PYCW	0.123 ± 0.002	0.475 ± 0.013	1.046 ± 0.018 ^a^	1.741 ± 0.013	2.786 ± 0.036	4.424 ± 0.037
Main effect, *p*-Values	0.830	0.608	0.002	0.170	0.276	0.400
Contrast, *p*-Values:						
T1 vs. T2	0.437	0.242	0.001	0.070	0.113	0.077
T1 vs. T3	0.651	0.181	0.000	0.868	0.420	0.908
T1 vs. T4	0.654	0.511	0.001	0.691	0.451	0.718
T4 vs. T5	0.545	0.430	0.974	0.073	0.159	0.562
T4 vs. T6	0.545	0.935	0.959	0.139	0.342	0.621
**Feed Conversion Ratio (FCR)**						
T1: CON	0.889 ± 0.021	1.541 ± 0.015 ^b^	1.753 ± 0.029 ^b^	1.674 ± 0.043	1.823 ± 0.047	2.115 ± 0.044 ^b^
T2: CON + YCWE	0.878 ± 0.017	1.468 ± 0.026 ^a^	1.591 ± 0.020 ^a^	1.625 ± 0.066	1.723 ± 0.027	2.022 ± 0.03 ^a^
T3: CON + PYCW	0.845 ± 0.006	1.428 ± 0.007 ^a^	1.567 ± 0.022 ^a^	1.574 ± 0.022	1.731 ± 0.025	2.016 ± 0.035 ^a^
T4: TOX	0.89 ± 0.016	1.503 ± 0.016 ^b^	1.667 ± 0.017 ^ab^	1.674 ± 0.036	1.899 ± 0.086	2.326 ± 0.055 ^c^
T5: TOX + YCWE	0.899 ± 0.012	1.518 ± 0.052 ^b^	1.624 ± 0.039 ^ab^	1.606 ± 0.016	1.808 ± 0.035	2.026 ± 0.036 ^b^
T6: TOX + PYCW	0.898 ± 0.008	1.482 ± 0.040 ^b^	1.616 ± 0.024 ^ab^	1.596 ± 0.072	1.763 ± 0.035	2.085 ± 0.045 ^b^
Main effect, *p*-Values	0.055	0.027	0.001	0.352	0.106	<0.001
Contrast, *p*-Values:						
T1 vs. T2	0.197	0.041	<0.001	0.431	0.069	0.039
T1 vs. T3	0.130	0.014	<0.001	0.092	0.385	0.022
T1 vs. T4	0.363	0.636	0.090	0.932	0.316	0.011
T4 vs. T5	0.942	0.711	0.862	0.111	0.301	0.000
T4 vs. T6	0.141	0.221	0.108	0.372	0.036	0.000

* Treatments description: T1 = Control diet with no supplement, no mycotoxin challenge (CON); T2 = T1 + YCWE (Mycosorb A+^®^, Alltech, Inc., Nicholasville, KY, USA); T3 = T1 + PYCW (Alltech, Inc., Nicholasville, KY, USA); T4 = Mycotoxin contaminated diet (TOX: 3.0 mg/kg DON; 2.17 mg/kg 3-ADON; 104 μg/kg T-2; 79 μg/kg ZEA); T5 = T4 + YCWE; T6 = T4 + PYCW. Supplements were used at 0.2 kg/T inclusion rate. ANOVA followed by Tukey’s multiple range test. Means within a column with no common letter indicate significant differences, *p* ≤ 0.05 (ANOVA followed by Tukey’s multiple range test).

**Table 2:** Comparison of the serum metabolites levels on day 21 and 42 of broilers fed a control diet or a *Fusarium*-mycotoxins contaminated diet, with or without supplementation of a yeast cell wall extract (YCWE) or postbiotic yeast cell wall-based blend (PYCW) added at an inclusion rate of 2.0 kg/T (*n* = 12).

Treatments *	Total Protein	Albumin	Albumin-to-Total Protein Ratio	Glucose	Creatinine	Uric Acid	Cholesterol
	g/L	g/L	Ratio	mg/DL	mg/DL	mg/DL	mg/DL
**Day 21**							
T1: CON	34.068 ± 0.962	17.299 ± 0.551	0.508 ± 0.005	282.86 ± 0.99 ^c^	1.17 ± 0.04	5.90 ± 0.44	76.90 ± 2.71 ^b^
T2: CON + YCWE	32.117 ± 0.839	16.209 ± 0.590	0.504 ± 0.008	239.65 ± 1.59 ^b^	1.11 ± 0.04	7.24 ± 0.52	71.63 ± 2.87 ^ab^
T3: CON + PYCW	32.725 ± 0.486	16.463 ± 0.392	0.503 ± 0.006	237.17 ± 1.82 ^b^	1.13 ± 0.02	7.44 ± 0.55	59.88 ± 3.39 ^a^
T4: TOX	34.104 ± 0.305	17.053 ± 0.227	0.501 ± 0.006	241.54 ± 7.71 ^b^	1.20 ± 0.04	6.78 ± 0.38	79.03 ± 1.90 ^b^
T5: TOX + YCWE	33.626 ± 0.387	17.299 ± 0.298	0.515 ± 0.006	210.90 ± 8.68 ^a^	1.31 ± 0.06	6.93 ± 0.48	73.07 ± 4.96 ^ab^
T6: TOX + PYCW	34.467 ± 0.291	17.880 ± 0.226	0.519 ± 0.006	200.50 ± 7.05 ^a^	1.18 ± 0.03	6.32 ± 0.37	58.87 ± 4.41 ^a^
Main effect, *p*-Values	0.057	0.060	0.182	<0.001	0.444	0.211	<0.001
Contrast, *p*-Values:							
T1 vs. T2	0.142	0.063	0.648	<0.001	0.366	0.166	0.194
T1 vs. T3	0.121	0.151	0.561	<0.001	0.459	0.144	0.001
T1 vs. T4	0.967	0.671	0.388	<0.001	0.722	0.147	0.527
T4 vs. T5	0.578	0.672	0.091	0.015	0.139	0.820	0.278
T4 vs. T6	0.673	0.156	0.028	0.001	0.756	0.399	0.001
**Day 42**							
T1: CON	33.562 ± 0.565 ^a^	17.071 ± 0.365 ^a^	0.509 ± 0.006	267.35 ± 4.31 ^c^	1.12 ± 0.02 ^b^	6.35 ± 0.24	89.90 ± 2.50
T2: CON + YCWE	33.897 ± 0.497 ^a^	17.077 ± 0.412 ^a^	0.504 ± 0.007	237.60 ± 5.72 ^b^	1.04 ± 0.02 ^ab^	5.72 ± 0.35	82.76 ± 3.74
T3: CON + PYCW	33.877 ± 0.803 ^a^	17.436 ± 0.575 ^a^	0.514 ± 0.007	244.51 ± 3.23 ^b^	0.98 ± 0.02 ^a^	6.00 ± 0.42	81.17 ± 3.31
T4: TOX	35.663 ± 0.319 ^b^	18.523 ± 0.137 ^b^	0.520 ± 0.006	225.30 ± 7.08 ^ab^	1.14 ± 0.02 ^b^	6.07 ± 0.35	90.57 ± 3.10
T5: TOX + YCWE	35.561 ± 0.188 ^b^	18.307 ± 0.196 ^b^	0.515 ± 0.007	211.07 ± 3.77 ^a^	1.14 ± 0.05 ^b^	6.63 ± 0.56	84.05 ± 5.63
T6: TOX + PYCW	35.683 ± 0.539 ^b^	18.528 ± 0.455 ^b^	0.519 ± 0.006	216.94 ± 4.16 ^a^	1.08 ± 0.01 ^ab^	6.95 ± 0.44	85.28 ± 6.71
Main effect, *p*-Values	0.004	0.009	0.384	<0.001	0.005	0.275	0.590
Contrast, *p*-Values:							
T1 vs. T2	0.651	0.991	0.545	<0.001	0.044	0.160	0.127
T1 vs. T3	0.672	0.507	0.549	<0.001	0.001	0.481	0.046
T1 vs. T4	0.006	0.010	0.193	<0.001	0.689	0.529	0.868
T4 vs. T5	0.890	0.693	0.582	0.095	0.952	0.410	0.323
T4 vs. T6	0.979	0.993	0.897	0.323	0.080	0.143	0.483

* Treatments description: T1 = Control with no supplement, no mycotoxin challenge (CON); T2 = T1 + YCWE; T3 = T1 + PYCW; T4 = Contaminated diet (TOX: 3.0 mg/kg DON; 2.17 mg/kg 3-ADON; 104 μg/kg T-2; 79 μg/kg ZEA); T5 = T4 + YCWE; T6 = T4 + PYCW. Means within a column bearing different superscripts differ significantly (*p* ≤ 0.05) for the main effects of treatments (ANOVA followed by Tukey’s multiple range test). Different superscript letters indicate significant difference between the treatments (*p* < 0.05).

**Table 3:** Comparison of the circulatory levels (expressed in U/L) in blood serum of four liver enzymes after 21 and 42 days in broilers, respectively, and fed a control diet or *Fusarium*-mycotoxins contaminated diet, with or without supplementation of a yeast cell wall extract (YCWE) or postbiotic yeast cell wall-based blend (PYCW) added at an inclusion rate of 2.0 kg/T (*n* = 12).

Treatments *	Day 21 (U/L)				Day 42 (U/L)			
	AST **	ALT	ALP	LDH	AST	ALT	ALP	LDH
T1: CON	72.4 ± 2.92 ^c^	7.06 ± 0.49 ^ab^	92.81 ± 3.57 ^b^	2140 ± 83	76.67 ± 2.47 ^c^	16.66 ± 0.71 ^d^	98.15 ± 7.39 ^bc^	2353 ± 99 ^a^
T2: CON + YCWE	53.5 ± 2.11 ^a^	10.47 ± 0.51 ^c^	79.05 ± 2.44 ^a^	2130 ± 62	72.39 ± 1.74 ^bc^	8.99 ± 0.45 ^ab^	82.30 ± 2.84 ^a^	2395 ± 79 ^ab^
T3: CON + PYCW	59.6 ± 2.58 ^a^	8.73 ± 0.67 ^bc^	81.32 ± 1.78 ^a^	2094 ± 71	61.05 ± 1.58 ^a^	8.24 ± 0.77 ^a^	94.55 ± 7.96 ^b^	2175 ± 14 ^a^
T4: TOX	70.9 ± 4.44 ^bc^	5.93 ± 0.40 ^a^	117.46 ± 2.47 ^c^	2233 ± 66	82.41 ± 1.37 ^d^	12.04 ± 0.86 ^bc^	121.72 ± 7.29 ^d^	2618 ± 66 ^b^
T5: TOX + YCWE	62.5 ± 3.42 ^abc^	7.59 ± 0.68 ^b^	111.05±8.53 ^c^	2198 ± 77	67.97 ± 2.02 ^ab^	13.89 ± 1.19 ^cd^	118.9 ± 10.73 ^d^	2715 ± 13 ^b^
T6: TOX + PYCW	66.7 ± 1.77 ^bc^	7.17 ± 0.42 ^b^	109.24 ± 11.78 ^bc^	2137 ± 10	72.90 ± 2.43 ^bc^	16.10 ± 0.49 ^d^	106.88 ± 3.13 ^c^	2441 ± 10 ^ab^
Main effect, *p*-Values	<0.001	<0.001	<0.001	0.813	<0.001	<0.001	0.005	0.013
Contrast, *p*-Values:								
T1 vs. T2	<0.001	<0.001	0.008	0.926	0.172	<0.001	0.043	0.728
T1 vs. T3	0.003	0.061	0.016	0.684	<0.001	<0.001	0.745	0.309
T1 vs. T4	0.774	0.095	<0.001	0.390	0.058	<0.001	0.036	0.030
T4 vs. T5	0.159	0.052	0.488	0.728	<0.001	0.225	0.831	0.533
T4 vs. T6	0.405	0.045	0.518	0.439	0.003	0.001	0.084	0.165

* Treatment description: T1 = Control with no supplement, no mycotoxin challenge (CON); T2 = T1 + YCWE; T3 = T1 + PYCW; T4 = Contaminated diet (TOX: 3.0 mg/kg DON; 2.17 mg/kg 3-ADON; 104 μg/kg T-2; 79 μg/kg ZEA); T5 = T4 + YCWE; T6 = T4 + PYCW. ** Measured liver enzymes: aspartate aminotransferase (AST); alanine aminotransferase (ALT); alkaline phosphatase (ALP); and lactate dehydrogenase (LDH). Means within a column bearing different superscripts letters differ significantly (*p* ≤ 0.05) for the main effects of treatments (ANOVA followed by Tukey’s multiple range test).

**Table 4:** Proportion of short-chain fatty acids (SCFA) in caecal digesta of after slaughter at 21 days in broilers fed a control diet or *Fusarium*-mycotoxins contaminated diet, with or without supplementation of a yeast cell wall extract (YCWE) or postbiotic yeast cell wall-based blend (PYCW) added at an inclusion rate of 2.0 kg/T (*n* = 16).

Treatments *	Acetate	Propionate	Iso Butyrate	Butyrate	Iso Valerate	Valerate	Total
	Proportion of SCFA (%)						(μmol/g)
T1: CON	0.861 ± 0.011 ^c^	0.065 ± 0.006	0.01 ± 0.000	0.051 ± 0.006 ^a^	0.006 ± 0.000 ^a^	0.005 ± 0.000 ^a^	172.41 ± 9.13
T2: CON + YCWE	0.833 ± 0.012 ^bc^	0.07 ± 0.004	0.01 ± 0.001	0.068 ± 0.006 ^ab^	0.008 ± 0.000 ^ab^	0.008 ± 0.002 ^ab^	172.56 ± 12.7
T3: CON + PYCW	0.79 ± 0.012 ^ab^	0.07 ± 0.005	0.014 ± 0.001	0.107 ± 0.008 ^cd^	0.008 ± 0.001 ^ab^	0.009 ± 0.001 ^b^	172.50 ± 8.87
T4: TOX	0.795 ± 0.012 ^ab^	0.085 ± 0.009	0.014 ± 0.001	0.081 ± 0.006 ^abc^	0.012 ± 0.001 ^b^	0.011 ± 0.001 ^b^	187.03 ± 7.03
T5: TOX + YCWE	0.799 ± 0.006 ^ab^	0.075 ± 0.005	0.013 ± 0.001	0.095 ± 0.005 ^bc^	0.010 ± 0.000 ^ab^	0.006 ± 0.000 ^a^	189.44 ± 9.32
T6: TOX + PYCW	0.779 ± 0.011 ^a^	0.068 ± 0.009	0.012 ± 0.002	0.123 ± 0.007 ^d^	0.008 ± 0.001 ^ab^	0.007 ± 0.000 ^a^	191.52 ± 6.86
Main effect, *p*-Values.	<0.001	0.494	0.216	<0.001	0.013	0.011	0.381
Contrast, *p*-Values:							
T1 vs. T2	0.095	0.630	0.887	0.085	0.256	0.083	0.991
T1 vs. T3	0.000	0.649	0.116	0.000	0.106	0.042	0.994
T1 vs. T4	0.000	0.068	0.059	0.003	0.000	0.001	0.255
T4 vs. T5	0.796	0.316	0.600	0.127	0.221	0.004	0.841
T4 vs. T6	0.304	0.108	0.218	0.000	0.018	0.013	0.710

* Treatments description: T1 = Control with no supplement, no mycotoxin challenge (CON); T2 = T1 + YCWE; T3 = T1 + PYCW; T4 = Contaminated diet (TOX: 3.0 mg/kg DON; 2.17 mg/kg 3-ADON; 104 μg/kg T-2; 79 μg/kg ZEA); T5 = T4 + YCWE; T6 = T4 + PYCW. Means within a column bearing different superscripts letters differ significantly (*p* ≤ 0.05) for the main effects of treatments (ANOVA followed by Tukey’s multiple range test).

**Table 5:** Proportions of short-chain fatty acids (SCFA) in caecal digesta of after slaughter at 42 days in broilers fed a control diet or *Fusarium*-mycotoxins contaminated diet, with or without supplementation of a yeast cell wall extract (YCWE) or postbiotic yeast cell wall-based blend (PYCW) added at an inclusion rate of 2.0 kg/T (*n* = 16).

Treatments *	Acetate	Propionate	Iso Butyrate	Butyrate	Iso Valerate	Valerate	Total
	Proportion of SCFA (%)						(μmol/g)
T1: CON	0.763 ± 0.015	0.145 ± 0.008	0.003 ± 0.003 ^a^	0.078 ± 0.009 ^a^	0.006 ± 0.001	0.003 ± 0.00	107.03 ± 4.01
T2: CON + YCWE	0.747 ± 0.007	0.175 ± 0.006	0.005 ± 0.005 _ab_	0.058 ± 0.005 ^a^	0.009 ± 0.000	0.004 ± 0.00	121.25 ± 5.96
T3: CON + PYCW	0.722 ± 0.004	0.182 ± 0.008	0.006 ± 0.006 ^b^	0.074 ± 0.006 ^a^	0.009 ± 0.000	0.004 ± 0.00	138.49 ± 6.41
T4: TOX	0.763 ± 0.017	0.143 ± 0.010	0.004 ± 0.004 ^ab^	0.074 ± 0.009 ^a^	0.010 ± 0.001	0.003 ± 0.00	118.17 ± 4.85
T5: TOX + YCWE	0.759 ± 0.008	0.14 ± 0.006	0.004 ± 0.004 ^ab^	0.084 ± 0.006 ^ab^	0.008 ± 0.001	0.003 ± 0.00	128.32 ± 7.92
T6: TOX + PYCW	0.727 ± 0.011	0.14 ± 0.015	0.003 ± 0.003 ^ab^	0.116 ± 0.010 ^b^	0.007 ± 0.000	0.003 ± 0.00	137.77 ± 12.23
Main effect, *p*-Values	0.062	0.056	0.013	0.015	<0.001	0.135	0.458
Contrast, *p*-Values:							
T1 vs. T2	0.412	0.068	0.028	0.121	0.065	0.467	0.241
T1 vs. T3	0.063	0.074	0.001	0.722	0.031	0.168	0.061
T1 vs. T4	0.982	0.914	0.188	0.760	0.015	0.843	0.333
T4 vs. T5	0.825	0.795	0.985	0.418	0.252	0.871	0.338
T4 vs. T6	0.088	0.827	0.679	0.000	0.088	0.541	0.067

* Treatments description: T1 = Control with no supplement, no mycotoxin challenge (CON); T2 = T1 + YCWE; T3 = T1 + PYCW; T4 = Contaminated diet (TOX: 3.0 mg/kg DON; 2.17 mg/kg 3-ADON; 104 μg/kg T-2; 79 μg/kg ZEA); T5 = T4 + YCWE; T6 = T4 + PYCW. Means within a column bearing different superscripts letter differ significantly (*p* ≤ 0.05) for the main effects of treatments (ANOVA followed by Tukey’s multiple range test).

**Table 6:** Measurement of the villi height (VH, expressed in μm), crypt depth (CD, expressed in μm) and villi height/crypt ratio (VH/CD) of duodenum, jejunum and ileum tissue samples (n = 16) collected on day 21 and 42 of broilers fed a *Fusarium* multi-mycotoxin challenge compared to a control diet, with or without supplementation of a yeast cell wall extract (YCWE) or postbiotic yeast cell wall-based blend (PYCW) at an inclusion rate of 2.0 kg/T.

Treatments *		Day 21 (μm)			Day 42 (μm)		
		Duodenum	Jejunum	Ileum	Duodenum	Jejunum	Ileum
T1: CON	VH	1682.9 ± 22.3 ^b^	1432.1 ± 45.9 ^b^	634.0 ± 12.3 ^b^	1922.7 ± 36.6 ^b^	1473.6 ± 54.9 ^b^	973.9 ± 16.5 ^b^
T2: CON + YCWE	VH	1823.8 ± 44.4 ^cd^	1680.3 ± 85.5 ^cd^	698.6 ± 36.3 ^bc^	1969.6 ± 55.1 ^b^	1475.8 ± 50.6 ^b^	1041.9 ± 16.1 ^bc^
T3: CON + PYCW	VH	2138.1 ± 25.9 ^e^	1856.8 ± 32.6 ^d^	719.7 ± 15.7 ^bc^	2305.3 ± 50.3 ^c^	1656.9 ± 34.0 ^c^	1041.3 ± 27.2 ^bc^
T4: TOX	VH	1483.5 ± 16.5 ^a^	1147.8 ± 24.8 ^a^	524.3 ± 13.2 ^a^	1602.8 ± 30.0 ^a^	1127.0 ± 39.8 ^a^	698.2 ± 24.2 ^a^
T5: TOX + YCWE	VH	1748.8 ± 17.4 ^bc^	1442.4 ± 32.6 ^b^	694.3 ± 24.7 ^bc^	1928.8 ± 21.9 ^b^	1126.8 ± 27.2 ^a^	777.7 ± 19.0 ^a^
T6: TOX + PYCW	VH	1890.4 ± 22.1 ^d^	1603.2 ± 34.2 ^bc^	748.2 ± 40.7 ^c^	2113.2 ± 82.2 ^bc^	1469.9 ± 28.6 ^b^	1092.4 ± 38.6 ^c^
Main effect, *p*-values		<0.001	<0.001	<0.001	<0.001	<0.001	<0.001
T1: CON	CD	233.4 ± 08.8 ^bc^	195.6 ± 8.34 ^b^	139.3 ± 7.3 ^ab^	332.1 ± 14.0 ^b^	287.5 ± 10.2 ^bc^	255.4 ± 11.7 ^b^
T2: CON + YCWE	CD	172.8 ± 09.5 ^a^	152.6 ± 5.7 ^a^	132.4 ± 6.6 ^a^	371.4 ± 15.7 ^b^	339.3 ± 18.0 ^c^	233.2 ± 9.5 ^b^
T3: CON + PYCW	CD	201.9 ± 12.0 ^ab^	178.8 ± 5.7 ^ab^	131.3 ± 7.1 ^a^	371.4 ± 14.6 ^b^	330.3 ± 17.3 ^bc^	238.0 ± 9.5 ^b^
T4: TOX	CD	247.1 ± 08.1 ^c^	189.9 ± 8.7 ^b^	165.6 ± 6.3 ^bc^	244.2 ± 08.7 ^a^	196.2 ± 8.5 ^a^	170.4 ± 7.5 ^a^
T5: TOX + YCWE	CD	239.6 ± 12.3 ^bc^	232.6 ± 11.34 ^c^	168.9 ± 9.3 ^bc^	360.9 ± 14.1 ^b^	275.1 ± 12.9 ^b^	231.1 ± 7.3 ^b^
T6: TOX + PYCW	CD	239.0 ± 10.5 ^bc^	188.6 ± 6.2 ^b^	171.8 ± 7.3 ^c^	350.6 ± 13.6 ^b^	306.2 ± 14.0 ^bc^	255.4 ± 13.0 ^b^
Main effect, *p*-values		<0.050	<0.001	< 0.001	<0.001	<0.001	<0.001
		**Ratio, Day 21**			**Ratio, Day 42**		
T1: CON	VH/CD	7.4 ± 0.3 ^ab^	7.5 ± 0.4 ^ab^	4.7 ± 0.2 ^bcd^	6.0 ± 0.3	5.2 ± 0.2 ^ab^	3.9 ± 0.2 ^ab^
T2: CON + YCWE	VH/CD	11.0 ± 0.6 ^c^	11.2 ± 0.7 ^c^	5.6 ± 0.5 ^cd^	5.4 ± 0.3	4.4 ± 0.2 ^a^	4.6 ± 0.2 ^b^
T3: CON + PYCW	VH/CD	11.3 ± 0.7 ^c^	10.6 ± 0.4 ^c^	5.7 ± 0.3 ^d^	6.3 ± 0.3	5.2 ± 0.2 ^ab^	4.4 ± 0.2 ^b^
T4: TOX	VH/CD	6.1 ± 0.3 ^a^	6.3 ± 0.3 ^a^	3.2 ± 0.2 ^a^	6.7 ± 0.2	6.1 ± 0.5 ^b^	4.3 ± 0.3 ^ab^
T5: TOX + YCWE	VH/CD	7.7 ± 0.4 ^ab^	6.5 ± 0.5 ^a^	4.3 ± 0.3 ^ab^	5.5 ± 0.2	4.2 ± 0.2 ^a^	3.4 ± 0.2 ^a^
T6: TOX + PYCW	VH/CD	8.2 ± 0.4 ^b^	8.6 ± 0.3 ^b^	4.4 ± 0.2 ^ab^	6.2 ± 0.4	5.0 ± 0.3 ^ab^	4.5 ± 0.3 ^b^
Main effect, *p*-values		<0.050	<0.010	<0.010	<0.026	<0.010	<0.006

* Treatments description: T1 = Control with no supplement, no mycotoxin challenge (CON); T2 = T1 + YCWE; T3 = T1 + PYCW; T4 = Contaminated diet (TOX: 3.0 mg/kg DON; 2.17 mg/kg 3-ADON; 104 μg/kg T-2; 79 μg/kg ZEA); T5 = T4 + YCWE; T6 = T4 + PYCW. Means within a column bearing different superscripts letters differ significantly (*p* ≤ 0.05) for the main effects of treatments (ANOVA followed by Tukey’s multiple range test).

**Table 7:** The EPEF values for broilers on a *Fusarium* multi-mycotoxin challenge compared to a control diet, with or without supplementation of a yeast cell wall extract (YCWE) or postbiotic yeast cell wall-based blend (PYCW) at an inclusion rate of 2.0 kg/T.

Treatments *	EPEF Values
T1: CON	206.32 ± 8.79 ^a^
T2: CON + YCWE	221.76 ± 6.57 ^a^
T3: CON + PYCW	223.74 ± 6.91 ^a^
T4: TOX	176.68 ± 8.59 ^b^
T5: TOX + YCWE	229.08 ± 5.36 ^a^
T6: TOX + PYCW	213.99 ± 8.72 ^a^
Main effect, *p*-Values	0.001
Contrast, *p*-Values:	
T1 vs. T2	0.146
T1 vs. T3	0.103
T1 vs. T4	0.011
T4 vs. T5	0.000
T4 vs. T6	0.003

* Treatments description: T1 = Control with no supplement, no mycotoxin challenge (CON); T2 = T1 + YCWE; T3 = T1 + PYCW; T4 = Contaminated diet (TOX: 3.0 mg/kg DON; 2.17 mg/kg 3-ADON; 104 μg/kg T-2; 79 μg/kg ZEA); T5 = T4 + YCWE; T6 = T4 + PYCW. Products were tested at the inclusion rate of 0.2% *w/w*. Means within a column bearing different superscripts letter differ significantly (*p* ≤ 0.05) for the main effects of treatments (ANOVA followed by Tukey’s multiple range test).

**Table 8:** Composition and the nutritive value of the experimental control diets in a three-phase feeding program for broilers.

Ingredients, %	Pre-Starter Diet	Starter Diet	Finisher Diet
	(0–14 Days)	(15–28 Days)	(29–42 Days)
Corn	52.17	56.48	60.90
Soybean meal (45%CP)	40.90	35.54	31.30
Palm oil	3.00	4.30	4.80
Mineral mixture *	1.50	1.50	1.50
Dicalcium phosphate	1.50	1.00	0.80
Common Salt	0.40	0.35	0.30
Vitamin Premix **	0.20	0.10	0.15
DL-Methionine	0.20	0.15	0.15
B-complex	0.05	0.10	0.10
Antibiotic ***	0.03	0.03	0.03
Coccidiostat	0.05	0.05	0.05
Dry Matter, %	90.10	89.52	90.54
Metabolizable energy, kcal/kg	2977	3076	3096
Crude protein, %	23.01	21.54	19.28
Calcium	1.29	1.24	1.12
Available Phosphorus	0.66	0.59	0.53
Digestible Methionine	0.54	0.54	0.54
Digestible Threonine	0.72	0.72	0.72
Digestible Lysine	1.08	1.08	1.08

* Mineral Mixture: Each 100 g contains 1.48 g magnesium oxide, 6.0 g ferrous sulfate, 0.05 g copper sulfate, 0.04 g manganese sulfate, 0.001 g potassium iodide, 1.0 g zinc sulfate, 17.09 g potassium chloride, and 0.001 g sodium selenite. ** Vitamin Premix: Each 100 g contains 0.165 g vitamin a/d3 (vitamin a-10,00,000 IU/g, vitamin d-200,000 IU/g), 0.103 g vitamin k3, 0.206 g thiamine mononitrate, 0.513 g riboflavin, 0.309 g pyridoxine hydrochloride, 0.31 mg cyanocobalamin, 0.103 g folic acid, 4.124 g niacin, 1.031 g calcium-d-pantothenate, 1.5 g biotin, 89.545 g maltodextrin. *** Oxytetracycline. The nutritive value of the feed component including Ca, P, protein, dry matter was sent to the Eurofin analytical Lab (Bangalore, Karnataka, India) for analytical values. The other components (ME, Methionine, Threonine and Lysine) are calculated values.

**Table 9:** Initial concentrations of *Fusarium* mycotoxins in fermented media and final concentration in the contaminated treatment diets measured by LC-MS/MS.

Contaminated Diet	Mycotoxin	Concentration	Final Dietary Concentration
		mg/kg	mg/kg
Basal toxin concentration of control diet	Aflatoxin B1	0.006	0.006
	Citrinin	0.047	0.047
	Cyclopiazonic acid	0.016	0.016
	Fumonisins (B1+B2+B3)	0.310	0.310
	Fusaric acid	0.257	0.257
	Fusarenon X	0.045	0.045
Wheat media inoculated with *Fusarium sporotrichoides* ITCC1894	Zearalenone	47.32	0.079
	Beauvericin	161.64	0.167
	T-2 toxin	9.715	0.104
	HT-2 toxin	73.31	0.076
	Neosolaniol	72.49	0.075
Rice media inoculated with *Fusarium culmorum* ITCC148	Deoxynivalenol	3601.25	3.001
	3-Acetyldeoxynivalenol	2613.98	2.178
	Fusaric acid	0.50	0.000
	Sterigmatocystin	4.04	0.003

## Data Availability

Not applicable.

## Supplementary Materials

The following supporting information can be downloaded at: https://www.mdpi.com/article/10.3390/toxins14050315/s1, Figure S1: Comparison of the internal organ weights (expressed as relative percentage to total body weight) after respectively 21 and 42 days in broilers fed a control diet or *Fusarium*-mycotoxins contaminated diet, with or without supplementation of a yeast cell wall extract (YCWE) or postbiotic yeast cell wall-based blend (PYCW) added at an inclusion rate of 2.0 kg/T; Figure S2: Effect of a *Fusarium* multi-mycotoxin challenge compared to a control diet on the caecal concentration of (a) acetate, (b) propionate, (c) butyrate, (d) isobutyrate, (e) valerate, (f) isovalerate short-chain fatty acids of broilers with or without the addition of a yeast cell wall extract (YCWE) or postbiotic yeast cell wall-based blend (PYCW); Figure S3: Histological observations of intestinal villi using light microscopy after staining of duodenum, jejunum and ileum small intestine tissue samples collected on day 21 for broilers fed *Fusarium* multi-mycotoxin challenge compared to a control diet with or without the addition of a yeast cell wall extract (YCWE) or postbiotic yeast cell wall-based blend (PYCW); Figure S4: Histological observations of intestinal villi using light microscopy after staining of duodenum, jejunum and ileum small intestine tissue samples collected on day 42 for broilers fed *Fusarium* multi-mycotoxin challenge compared to a control diet, with or without the addition of a yeast cell wall extract (YCWE) or postbiotic yeast cell wall-based blend (PYCW); Table S1: Monitoring of the gut intestinal pH after respectively 21 and 42 days in broilers fed a control diet or *Fusarium*-mycotoxins contaminated diet, with or without supplementation of a yeast cell wall extract (YCWE) or postbiotic yeast cell wall-based blend (PYCW) added at an inclusion rate of 2.0 kg/T; Table S2: Goblet cell count in the small intestine of broilers fed a basal diet or *Fusarium* contaminated diet supplemented, with or without supplementation of a yeast cell wall extract-based product (YCWE) or postbiotic yeast cell wall-based product (PYCW) at an inclusion rate of 2.0 kg/T.
