# Supplementary material for: Impact of a Natural Fusarial Multi-Mycotoxin Challenge on Broiler Chickens and Mitigation Properties Provided by a Yeast Cell Wall Extract and a Postbiotic Yeast Cell Wall-Based Blend
Source: Toxins (Basel). 2022 Apr 28;14(5):315. doi: 10.3390/toxins14050315 (PMC9145611; doi:10.3390/toxins14050315)
Supplement: Supplementary file 1 [file toxins-14-00315-s001.zip › toxins-1659474-supplementary.pdf]

toxins

MDPI

Supplementary section

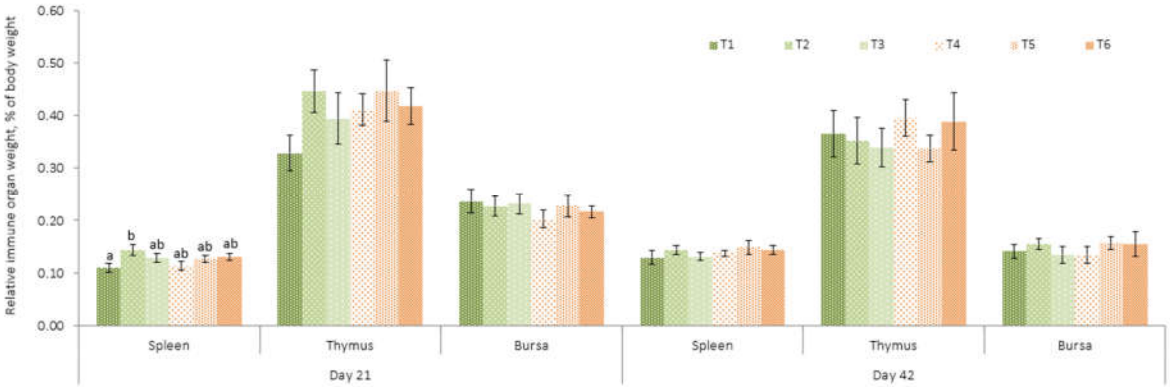

Figure S1. Comparison of the internal organ weights (expressed as relative percentage to total body weight) after respectively 21 and 42 days in broilers fed a control diet or *Fusarium*-mycotoxins contaminated diet, with or without supplementation of a yeast cell wall extract (YCWE) or postbiotic yeast cell wall-based blend (PYCW) added at an inclusion rate of 2.0 kg/T. T1 = Control with no supplement and no mycotoxin challenge (CON); T2 = T1 + YCWE; T3 = T1 + PYCW; T4 = Contaminated diet (TOX: 3.0 mg/kg DON, 0.1 mg/kg T-2, 0.079 mg/kg ZEA); T5 = T4 + YCWE; T6 = T4 + PYCW. Different superscript letters above the bars indicate significant differences between treatments, $P \leq 0.05$ (ANOVA followed by Tukey's multiple range test).

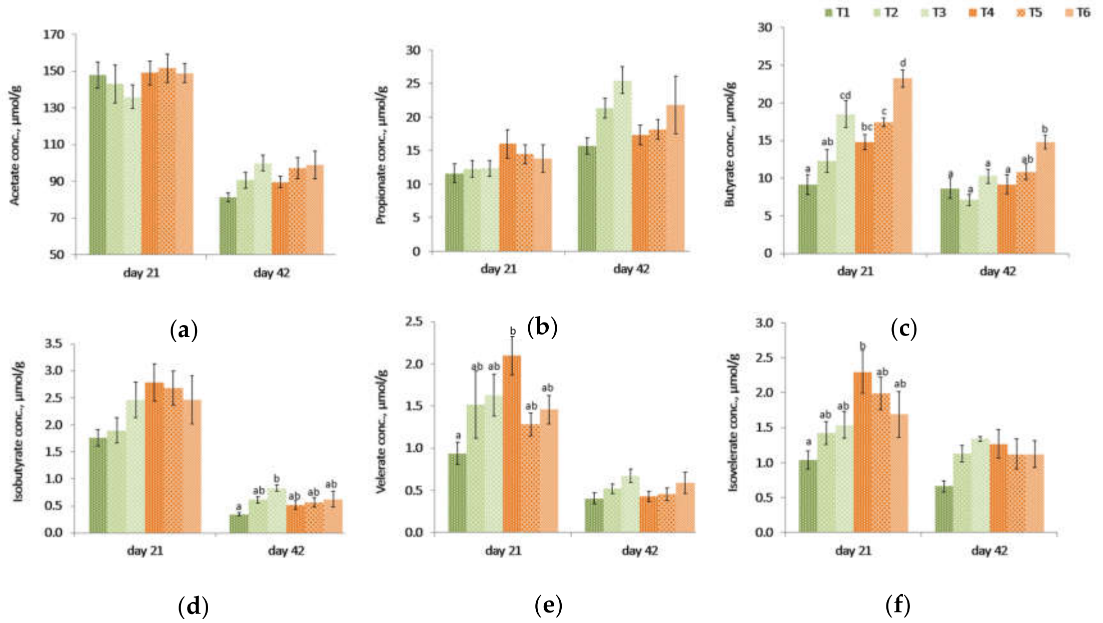

Figure S2. Effect of a *Fusarium* multi-mycotoxin challenge compared to a control diet on the caecal concentration of (a) acetate, (b) propionate, (c) butyrate, (d) isobutyrate, (e) valerate, (f) isovalerate short-chain fatty acids of broilers with or without the addition of a yeast cell wall extract (YCWE) or postbiotic yeast cell wall-based blend (PYCW) (mean ± SE). T1 = Control with no supplement and no mycotoxin challenge (CON); T2 = T1 + YCWE; T3 = T1 + PYCW; T4 = Contaminated diet (TOX: 3.0 mg/kg DON, 0.1 mg/kg T-2, 0.079 mg/kg ZEA); T5 = T4 + YCWE; T6 = T4 + PYCW. Different superscript letters above the bars indicate significant differences between treatments, $P \leq 0.05$ (ANOVA followed by Tukey's multiple range test).

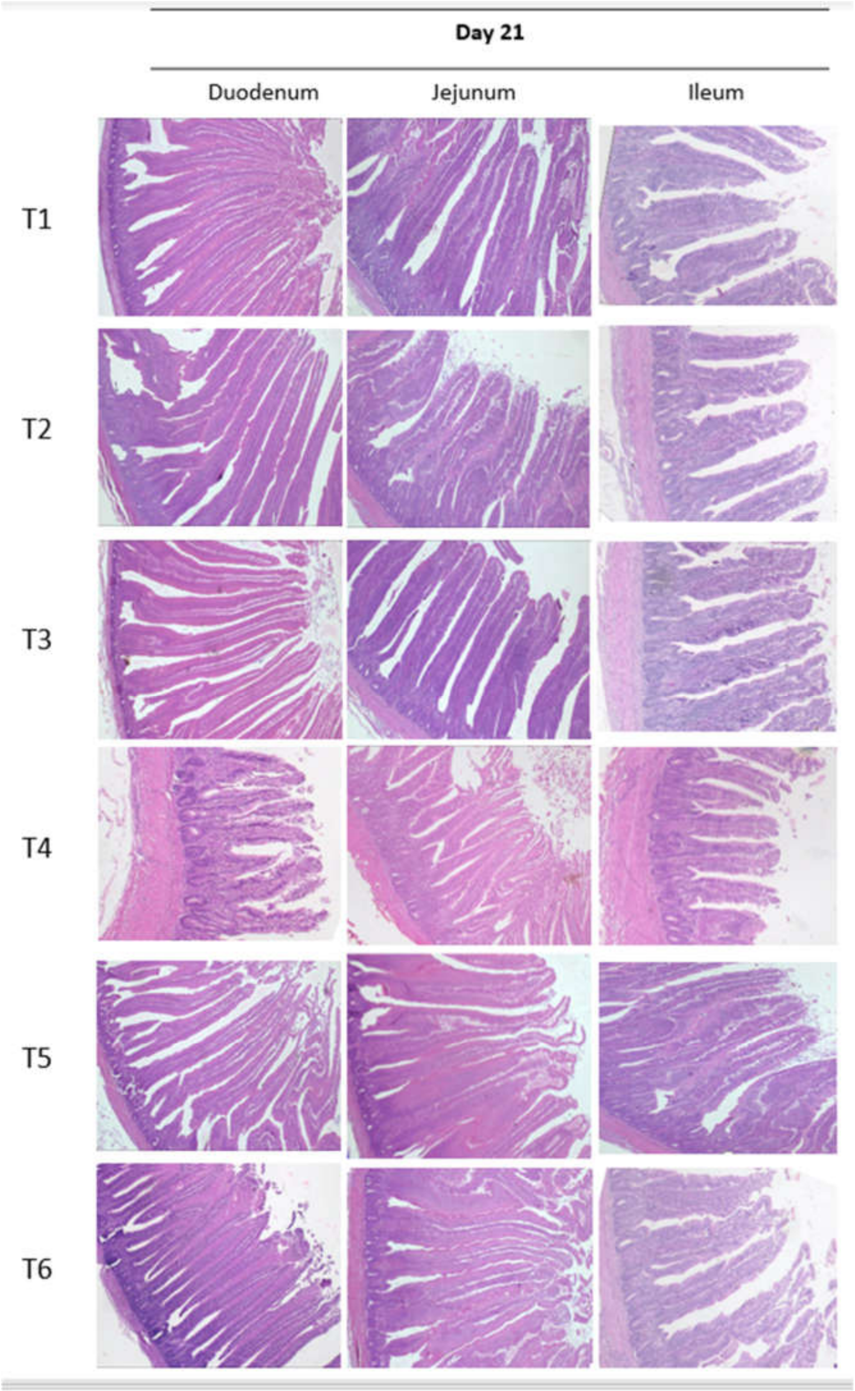

Figure S3. Histological observations of intestinal villi using light microscopy after staining of duodenum, jejunum and ileum small intestine tissue samples collected on day 21 for broilers fed *Fusarium* multi-mycotoxin challenge compared to a control diet with or without the addition of a yeast cell wall extract (YCWE) or postbiotic yeast cell wall-based blend (PYCW). T1 = Control with no supplement and no mycotoxin challenge (CON); T2 = T1 + YCWE; T3 = T1 + PYCW; T4 = Contaminated diet (TOX: 3.0 mg/kg DON; 2.17 mg/kg 3-ADON; 104 µg/kg T-2; 79 µg/kg ZEA); T5 = T4 + YCWE; T6 = T4 + PYCW.

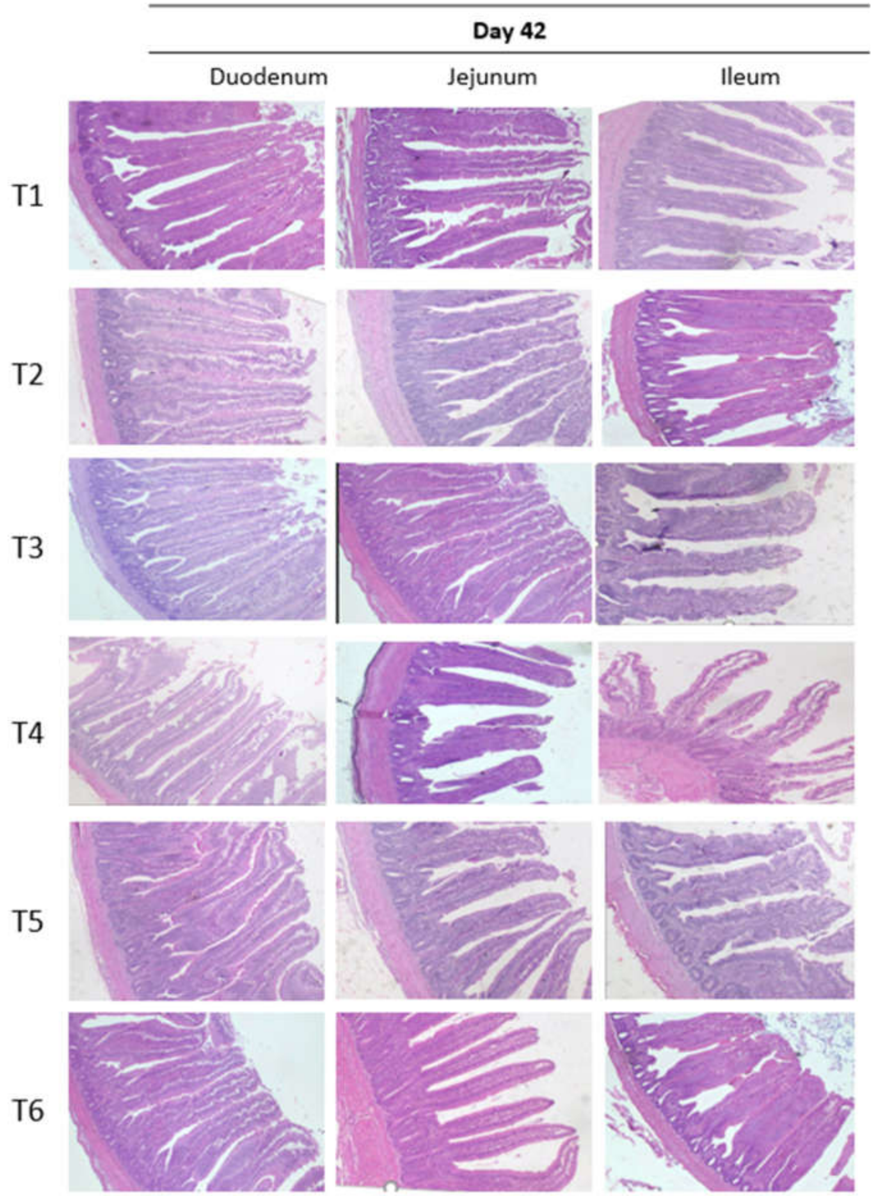

Figure S4. Histological observations of intestinal villi using light microscopy after staining of duodenum, jejunum and ileum small intestine tissue samples collected on day 42 for broilers fed *Fusarium* multi-mycotoxin challenge compared to a control diet, with or without the addition of a yeast cell wall extract (YCWE) or postbiotic yeast cell wall-based blend (PYCW). T1 = Control with no supplement and no mycotoxin challenge (CON); T2 = T1 + YCWE; T3 = T1 + PYCW; T4 = Contaminated diet (TOX: 3.0 mg/kg DON; 2.17 mg/kg 3-ADON; 104 µg/kg T-2; 79 µg/kg ZEA); T5 = T4 + YCWE; T6 = T4 + PYCW.

Table S1. Monitoring of the gut intestinal pH after respectively 21 and 42 days in broilers fed a control diet or *Fusarium*-mycotoxins contaminated diet, with or without supplementation of a yeast cell wall extract (YCWE) or postbiotic yeast cell wall-based blend (PYCW) added at an inclusion rate of 2.0 kg/T.

**Treatments***	**pH at Day 21**			**pH at Day 42**		
	**Duodenum**	**Jejunum**	**Ileum**	**Duodenum**	**Jejunum**	**Ileum**
T1: CON	6.15 ± 0.02	5.82 ± 0.07	5.83 ± 0.08	5.93 ± 0.03	5.72 ± 0.03	6.09 ± 0.08[ab]
T2: CON + YCWE	6.03 ± 0.04	5.68 ± 0.04	5.69 ± 0.09	5.87 ± 0.07	5.83 ± 0.05	6.04 ± 0.09[ab]
T3: CON + PYCW	6.03 ± 0.03	5.62 ± 0.05	5.68 ± 0.09	6.02 ± 0.04	5.76 ± 0.10	5.85 ± 0.08[a]
T4: TOX	6.07 ± 0.02	5.69 ± 0.04	5.73 ± 0.07	5.98 ± 0.08	5.88 ± 0.05	6.20 ± 0.06[b]
T5: TOX + YCWE	6.05 ± 0.02	5.64 ± 0.05	5.68 ± 0.08	6.07 ± 0.04	5.89 ± 0.04	6.34 ± 0.06[b]
T6: TOX + PYCW	6.03 ± 0.03	5.71 ± 0.03	5.57 ± 0.09	6.10 ± 0.03	5.86 ± 0.05	6.07 ± 0.09[ab]
Main effect, *P*-Values	0.156	0.164	0.522	0.053	0.277	**0.003**
Contrast, *P*-Values:						
T1 vs T2	0.053	0.132	0.318	0.474	0.135	0.653
T1 vs T3	**0.018**	**0.035**	0.285	0.106	0.689	**0.048**
T1 vs T4	0.062	0.137	0.423	0.585	**0.011**	0.318
T4 vs T5	0.662	0.535	0.653	0.361	0.864	0.155
T4 vs T6	0.349	0.708	0.198	0.187	0.800	0.274

*Treatments description: T1 = Control with no supplement, no mycotoxin challenge (CON); T2 = T1 + YCWE; T3 = T1 + PYCW; T4 = Contaminated diet (TOX: 3.0 mg/kg DON, 0.1 mg/kg T-2, 0.079 mg/kg ZEA); T5 = T4 + YCWE; T6 = T4 + PYCW. Means within a column bearing different superscripts differ significantly ($P \leq 0.05$) for the main effects of treatments (ANOVA followed by Tukey's multiple range test).

Table S2. Goblet cell count in the small intestine of broilers fed a basal diet or *Fusarium* contaminated diet supplemented, with or without supplementation of a yeast cell wall extract-based product (YCWE) or postbiotic yeast cell wall-based product (PYCW) at an inclusion rate of 2.0 kg/T (n=12).

**Treatments***	**Counts, Day 21**			**Counts, Day 42**		
	Duodenum	Jejunum	Ileum	Duodenum	Jejunum	Ileum
T1: CON	6.43 ± 0.37[ab]	6.50 ± 0.77	6.62 ± 0.35[ab]	8.06 ± 0.43[a]	7.81 ± 0.34	6.87 ± 0.59[a]
T2: CON + YCWE	8.06 ± 1.06[a]	7.93 ± 0.87	7.93 ± 0.34[b]	8.87 ± 0.22[a]	7.43 ± 0.35	7.75 ± 0.41[ab]
T3: CON + PYCW	8.75 ± 1.16[a]	8.37 ± 0.59	8.25 ± 0.35[b]	10.68 ± 0.31[b]	7.93 ± 0.40	9.18 ± 0.43[b]
T4: TOX	4.56 ± 0.4[b]	6.25 ± 0.44	5.31 ± 0.61[a]	8.18 ± 0.2[a]	7.31 ± 0.40	6.43 ± 0.31[a]
T5: TOX + YCWE	8.12 ± 0.76[a]	7.56 ± 0.57	6.81 ± 0.56[ab]	11.12 ± 0.41[b]	8.43 ± 0.34	7.62 ± 0.45[ab]
T6: TOX + PYCW	8.31 ± 0.43[a]	7.93 ± 0.46	6.62 ± 0.61[ab]	10.37 ± 0.35[b]	8.68 ± 0.28	8.93 ± 0.26[b]
Main effect, *P*-Values	**0.002**	0.123	**0.001**	**<0.001**	**0.052**	**<0.001**
Contrast, *P*-Values:						
T1 vs T2	0.140	0.117	0.061	0.090	0.460	0.152
T1 vs T3	**0.037**	0.052	**0.021**	**0.000**	0.805	**0.000**
T1 vs T4	0.089	0.784	0.061	0.793	0.325	0.472
T4 vs T5	**0.002**	0.151	**0.033**	**0.000**	**0.029**	0.053
T4 vs T6	**0.001**	0.066	0.061	**0.000**	**0.008**	**0.000**

*Treatments description: T1 = Control with no supplement, no mycotoxin challenge (CON); T2 = T1 + YCWE; T3 = T1 + PYCW; T4 = Contaminated diet (TOX: 3.0 mg/kg DON, 0.1 mg/kg T-2, 0.079 mg/kg ZEA); T5 = T4 + YCWE; T6 = T4 + PYCW. Means within a column bearing different superscripts differ significantly ($P \leq 0.05$) for the main effects of treatments (ANOVA followed by Tukey's multiple range test).
